# LncRNA CARMN inhibits abdominal aortic aneurysm formation and vascular smooth muscle cell phenotypic transformation by interacting with SRF

**DOI:** 10.1007/s00018-024-05193-4

**Published:** 2024-04-10

**Authors:** Shenrong Liu, Haobin Zhou, Dunzheng Han, Haoyu Song, Yuanqing Li, Shangfei He, Yipeng Du, Kai Wang, Xingfu Huang, Xin Li, Zheng Huang

**Affiliations:** 1 Department of Cardiology, The Key Laboratory of Advanced Interdisciplinary Studies Center, The First Affiliated Hospital of Guangzhou Medical University, Guangzhou, 510120 Guangdong China; 2 Wards of Cadres, Zhuhai People’s Hospital (Zhuhai Hospital Affiliated With Jinan University), Zhuhai, 519000 China; 3 Department of Cardiovascular Surgery, The Key Laboratory of Advanced Interdisciplinary Studies Center, The First Affiliated Hospital of Guangzhou Medical University, Guangdong, 510120 China; 4 Department of Cardiology, Nanfang Hospital, Southern Medical University, Guangzhou, 510400 Guangdong China; 5 Department of Emergency Medicine, Guangdong Provincial People’s Hospital (Guangdong Academy of Medical Sciences), Southern Medical University, Guangzhou, 510400 Guangdong China

**Keywords:** CARMN, Long noncoding RNA, Aneurysm, Vascular smooth muscle cell, SRF

## Abstract

Phenotypic transformation of vascular smooth muscle cells (VSMCs) plays a crucial role in abdominal aortic aneurysm (AAA) formation. CARMN, a highly conserved, VSMC-enriched long noncoding RNA (lncRNA), is integral in orchestrating various vascular pathologies by modulating the phenotypic dynamics of VSMCs. The influence of CARMN on AAA formation, particularly its mechanisms, remains enigmatic. Our research, employing single-cell and bulk RNA sequencing, has uncovered a significant suppression of CARMN in AAA specimens, which correlates strongly with the contractile function of VSMCs. This reduced expression of CARMN was consistent in both 7- and 14-day porcine pancreatic elastase (PPE)-induced mouse models of AAA and in human clinical cases. Functional analyses disclosed that the diminution of CARMN exacerbated PPE-precipitated AAA formation, whereas its augmentation conferred protection against such formation. Mechanistically, we found CARMN's capacity to bind with SRF, thereby amplifying its role in driving the transcription of VSMC marker genes. In addition, our findings indicate an enhancement in CAMRN transcription, facilitated by the binding of NRF2 to its promoter region. Our study indicated that CARMN plays a protective role in preventing AAA formation and restrains the phenotypic transformation of VSMC through its interaction with SRF. Additionally, we observed that the expression of CARMN is augmented by NRF2 binding to its promoter region. These findings suggest the potential of CARMN as a viable therapeutic target in the treatment of AAA.

**Graphical abstract:**

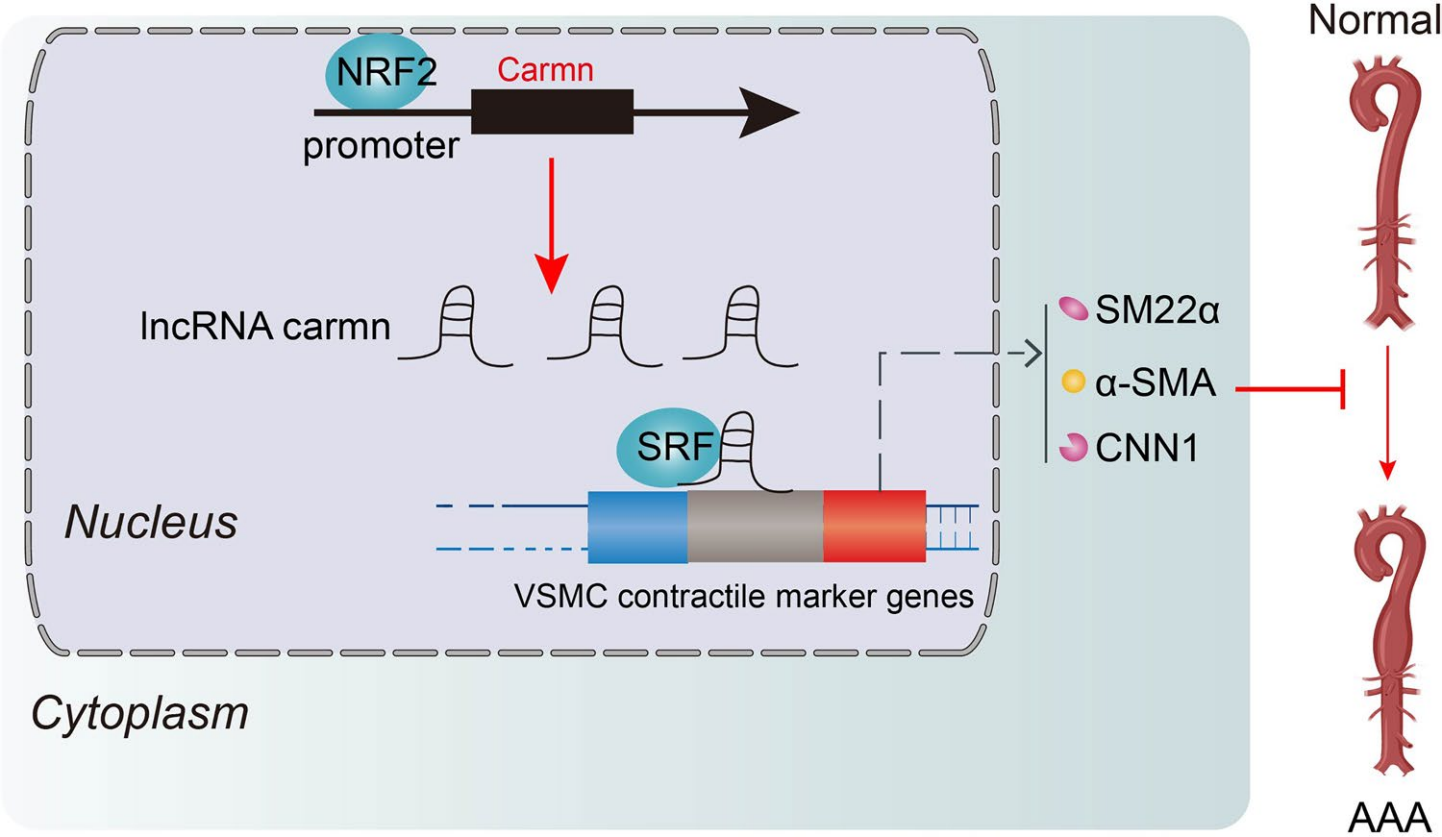

**Supplementary Information:**

The online version contains supplementary material available at 10.1007/s00018-024-05193-4.

## Introduction

Abdominal aortic aneurysm (AAA) represents a critical health risk, characterized by the gradual expansion and potential rupture of the abdominal aorta’s vascular wall [1]. Despite significant advancements in open and endovascular repair surgeries, there remains a substantial gap in effective pharmacological treatments for AAA patients not suitable for surgical intervention [2]. Consequently, deciphering the underlying molecular mechanisms and pinpointing therapeutic targets are of paramount importance in the quest for effective AAA treatment.

Smooth muscle cells (SMCs) exhibit remarkable diversity across different organs. SMC differ morphologically and functionally at vascular, gastrointestinal, respiratory, and urogenital tracts [3, 4]. Lee et al. reveals significant differences in cellular expression between human and mouse SMCs, including vascular SMCs and non-vascular SMCs [5]. These differences manifest across various organs and are dependent on cell type, with notable heterogeneity seen in specific regions like the aorta and non-vascular SMCs of the esophagus, bladder, and stomach [5]. Vascular smooth muscle cells (VSMCs), residing in the aortic media, exhibit the ability to oscillate between a contractile (or differentiated) phenotype and a synthetic (or dedifferentiated) phenotype [6]. In their contractile state, VSMCs are pivotal for maintaining blood vessel functionality, regulating vascular tone, blood pressure, and diameter [7]. When stimulated by pathological factors, VSMCs transition to the synthetic phenotype with augmented proliferative and migratory capabilities, excessive extracellular matrix component secretion, and diminished contractile marker expression [8]. This transformation from the contractile state to the synthetic state, also termed phenotypic transformation, is observed in many vascular diseases, including AAA [9]. Consequently, modulating VSMC phenotypic transformation presents a novel therapeutic approach for AAA, though the mechanisms driving this transformation during AAA developments are still largely unexplored.

Long noncoding RNAs (lncRNAs), characterized as RNA molecules exceeding 200 nucleotides without protein-coding potential, exhibit a high degree of tissue-specific expression [10]. LncRNAs appear to be highly tissue specific in expression and can regulate gene transcription, RNA and protein stability, and translation and posttranslational modifications [10]. Consequently, lncRNAs are increasingly recognized as both biomarkers and regulatory agents in a variety of diseases and pathophysiological conditions [11–13]. Recent evidence has highlighted the significant role of lncRNAs in modulation of VSMC functionality, contributing to various cardiovascular diseases, including atherogenesis [14, 15], hypertension [16], pulmonary arterial hypertension [17] and vascular remodeling [18]. LncRNAs are increasingly implicated in the formation and progression of AAA. Previous researches have identified specific lncRNAs, such as H19 [19], PVT1 [20] and LUCAT1 [21], as key contributors to AAA progression by influencing VSMC proliferation and apoptosis. However, a notable limitation in the clinical application of these lncRNAs arises from their partial presence in SMCs within the human body and their lack of cell specificity. This limitation underscores the need for more targeted IncRNA-based interventions in AAA treatment.

Cardiac mesoderm enhancer-associated noncoding RNA (CARMN) stands out as a notably conserved lncRNA predominantly located in SMCs [22]. Known for hosting miR143/145, CARMN mirrors their function in sustaining the contractile phenotype of VSMCs [23]. Growing evidence has demonstrated that CARMN participates in vascular diseases, including atherosclerosis [24, 25] and neointima formation [22]. Notably, reduced levels of CARMN were linked to advanced atherosclerotic lesions, with its knockdown in mice promoting a shift to a proliferative VSMC phenotype, thereby accelerating atherosclerotic [24]. CARMN exhibits the ability to bind to Myocardin (MYOCD), thereby enhancing the MYOCD-SRF (serum response factor) interaction and amplifying the transcriptional activity of contractile markers [22]. Additionally, CARMN’s interaction with SRF further facilitates SRF’s binding to target gene promoters, crucial for maintaining VSMC homeostasis [25]. Despite these insights, the role of CARMN in AAA formation remains an unexplored territory.

In our study, we hypothesized that CARMN might exert regulatory control over the VSMC phenotype, thereby influencing the development of AAA. We performed single-cell RNA sequencing to delineate the expression profile of CARMN in human AAA tissues. We furthered our investigation by manipulating CARMN levels—both its knockdown and overexpressing—in a porcine pancreatic elastase (PPE)-induced AAA model. This approach allowed us to gain deeper functional insights into CARMN’s role in AAA formation. Moreover, we investigated the downstream and upstream mechanisms of CARMN during AAA formation. These findings may provide new therapeutic methods for AAA treatment.

## Materials and methods

### Single-cell RNA sequencing using 10 × genomics chromium

To capture single cells, we used the 10 × Genomics Single Cell 3’ v3 kit. Subsequently, cDNA libraries were created from both normal abdominal aortic and AAA tissues, following the instructions provided by the manufacturer. The resulting libraries were then pooled and sequenced using the Illumina NovaSeq 6000 platform.

### Raw data preprocessing

The sequenced reads obtained from the platform underwent demultiplexing and were aligned with the GRCh38 human reference genome using CellRanger (7.0.1). Unique molecule identifiers (UMIs) were utilized to count and generate digital expression matrices for each gene. Cell barcodes were filtered by CellRanger. In summary, an expression for each sample was generated, detailing the UMI count per cell for each gene, which was imported as 10 × data object.

### Quality control (QC) and normalization

Data analysis was carried out using Seurat (4.0.2), an R package used for analysis of single-cell RNA sequencing data. During quality control, low-quality cells were excluded based on criteria such as the UMI counts, the number of expressed genes and the fraction of mitochondrial transcripts. Then, the count matrix of the remaining cells was subjected to scaling and normalization using Seurat's ScaleData and NormalizeData function, respectively. The top 2000 highly variable features were identified using the FindVariableFeatures function. Furthermore, the normalized data were scaled using the ScaleData function.

### Dataset integration, unsupervised clustering, marker identification, and cell type annotation

The identified highly variable genes were utilized to conduct principal component analysis (PCA). To mitigate batch effects and integrate single-cell RNA-seq data from both normal abdominal aortic and AAA samples, we utilized the harmony R package. Leveraging the top 20 principal components, an unsupervised cell clustering analysis was performed using Seurat's FindNeighbors and FindClusters functions. To visualize the data, we employed t-distributed stochastic neighbor embedding (t-SNE) to project cells onto a 2D space based on aligned canonical correlation analysis. To identify cluster-specific genes, we utilized Seurat's FindAllMarkers function and employed a Wilcoxon rank sum test. The marker genes were determined by setting the parameters as follows: min.pct = 0.1, adjusted p values < 0.05, and an average log2-fold change (log_2_fc) > 0.25 [26, 27]. Cell types were manually annotated based on their markers. Additionally, the activity of specific gene sets in each cell was quantified using the AddModuleScore function in Seurat.

### Bulk RNA sequencing data processing and analysis

The transcriptome sequencing datasets (GSE197748 and GSE226736) were downloaded from the GEO database (https://www.ncbi.nlm.nih.gov/geo/query/acc.cgi?acc=GSE197748, https://www.ncbi.nlm.nih.gov/geo/query/acc.cgi?acc=GSE226736). The raw data were read by the oligo package and standardized and reannotated using the rma method. Differentially expressed genes (DEGs) were identified using the limma package with the standards adjusted *p* values < 0.05 and |log2FC|> 1.5. KEGG and GO function enrichment analyses were conducted using the R package "clusterProfiler". The genomic information of *Carmn* and mRNA was obtained through the Metascape database. The interaction networks between proteins were acquired by the GeneMANIA database and the String database.

### Experimental animals

Healthy male C57BL/6 J mice, aged 8 to 12 weeks, were sourced from Guangzhou Medical University. All mice were housed under pathogen-free conditions featuring a 12-h dark/light cycle with fluorescent lighting, at a humidity level around 55%, and a temperature range of 18–22 °C. The mice had unrestricted access to food and purified water. The experiments conducted on animals were approved by the Chinese Academy of Basic Medical Sciences and the Animal Care and Use Committee of Guangzhou Medical University and abided by the guidelines of the Animal Research Advisory Committee of the National Institutes of Health.

### PPE-induced AAA models

The PPE-induced AAA model was established according to previous methods [28]. Briefly, each mouse was anesthetized through intraperitoneal injection of a mixture of xylazine (5 mg/kg) and ketamine (100 mg/kg). Adequate anesthesia was confirmed through the disappearance of the pedal withdrawal reflex. A midline abdominal incision was made to fully expose the infrarenal abdominal aorta up to the bifurcation. The sponge soaked with PPE (0.2 U) was covered onto the abdominal aorta for 5 min. The control group mice received saline in sham operations. The residual PPE in the abdominal cavity was washed with physiological saline, the incision was sutured. The mice were euthanized after 7 days or 14 days of feeding with normal chow for the next experiment.

### Adeno-associated virus (AAV) infection in mice

Murine Carmn-overexpressing adeno-associated virus serotype 9 (AAV9) and Carmn-knockdown AAV9 were manufactured by GeneChem (China). The transfection process was carried out by injecting AAV9 into the mouse tail vein according to previous methods [29]. The amount of virus used for transfection was 1 × 10^11^ vector genomes. The transfection effect was verified 30 days after injection.

### Quantification of aneurysm formation

Initially, the mice were perfused with 10% formaldehyde at physiological pressure via a pinhole on the left ventricle. Subsequently, the aorta was extracted and photographed using a digital camera to record changes in its diameter. The maximum diameter of the dilated segment of the infrarenal vessel was measured with Image-Pro Plus software (Media Cybernetics, USA). Based on the definition of human aneurysm established in previous studies [30], aneurysm formation was defined by at least a 50% increase in infrarenal aortic diameter in the PPE-treated mice compared with the saline-treated mice. In cases where mice died during the experiment, necropsies were conducted. AAAs were assessed by two investigators blinded to the experimental treatment, with at least thrice for accuracy.

### Verhoeff's van Gieson (VVG) staining

Tissue sections were prepared in accordance with previously established methods [30]. Five-micron-thick serial sections were stained using the Verhoeff Elastic Fiber Staining Kit (Servicebio, China). We graded the degree of elastin degradation according to the scoring standards of previous studies [31]: Grade I, elastic fibers are arranged regularly without breakage or degradation; Grade II, elastic fibers are slightly broken; Grade III, denotes severe damaged to elastic fibers, though not completely broken; and Grade IV represents aortic rupture.

### RNA extraction and qPCR

Total RNA from cells or tissues was extracted using TRIzol reagent (Invitrogen, Thermo Fisher Scientific, USA) and its concentration was determined using a NanoDrop Lite spectrophotometer (Thermo Fisher Scientific, USA). With the SYBR Premix Ex Taq Kit (TaKaRa Biotechnology, China), quantitative polymerase chain reaction (qPCR) was carried out on a QuantStudio 5 real-time fluorescent qPCR system (Applied Biosystems, Thermo Fisher Scientific, USA). The relative mRNA expression was measured by normalizing the internal reference (GAPDH in this study). The primer sequences are listed in Supplemental Table 1.

### Western blotting assay

The pretreated mouse aortic tissue was placed on ice, and then, the protein was extracted using RIPA buffer [consisting of Tris–HCl (25 mM, pH 7.6), NaCl (150 mM), NP-40 (1%), sodium deoxycholate (1%), and SDS (0.1%)]. The protein extracts were segregated on SDS‒PAGE gels and transferred to polyvinylidene fluoride (PVDF) blotting membranes. Initially, the membranes were incubated with primary antibodies overnight at 4 °C and secondary antibodies for 2 h at room temperature. Enhanced chemiluminescence reagent (RPN2235; GE-Healthcare Life Sciences, UK) was used for exposure imaging, which was repeated at least 5 times. Total protein was quantified using ImageJ software (National Institutes of Health, USA), with EIF5 or GAPDH serving as the endogenous normalization controls. The antibody information is shown in Supplemental Table 2.

### Immunofluorescence staining

After fixation in 4% paraformaldehyde, mouse aortic samples were prepared into five micrometer thick paraffin sections at intervals of approximately 500 µm, followed by dewaxing in water. For immunofluorescence staining, the sections were then subjected to antigen retrieval by soaking in 10 mmol/L sodium citrate buffer (pH 6.0) and boiling in a microwave oven. Next, we blocked the sections with 1% bovine serum and incubated them with primary antibodies. After secondary antibody incubation, the sections were incubated with DAPI (Beyotime, China). The slides were visualized with a Leica (TCS Sp8, Germany) confocal microscope. Supplemental Table 3 displays the information on the antibodies used in this study.

### RNA fluorescence in situ hybridization

Hybridization was carried out on the sections using hybridization buffer (RiboBio, China) and incubated them with a labeled CARMN probe overnight. Post-hybridization, the sections were purified using SSC and subsequently incubated with both primary antibody and secondary antibodies. The final step involved incubation in DAPI. The sequences of the human and mouse CARMN probes are detailed in Supplemental Table 4.

### Cell isolation and transfection

Primary aortic VSMCs were extracted using the enzymatic digestion method from the experimental mouse [32]. Briefly, the aorta was separated from the mouse, and the surrounding adipose tissue was removed. After isolation from the mouse, the aorta was longitudinally incised to expose the intima layer. The tissue was cut into 1 × 1 mm pieces and disintegrated in Hank's balanced salt solution (HBSS; Gibco, USA) with 0.744 U/ml elastase (Sigma, USA), 1 mg per ml collagenase type II (Sigma, USA) and 1 mg per ml trypsin inhibitor (Sigma, USA) for two hours at 37 °C. Following digestion, an equal volume of Dulbecco’s Modified Eagle’s Medium (DMEM) with 20% fetal bovine serum (FBS) was added to halt the reaction. The cells were harvested by centrifugation, then cultured in DMEM with 20% FBS. After 48 h to allow for adherence and initial growth, the medium was replaced with DMEM containing 10% FBS.

Before transfection, adherent VSMCs were inoculated into 6-well plates, cultured to 70–80% confluence, transfected with siRNA-Carmn, control siRNA, oe-Carmn, or control plasmid, and further cultured in a constant temperature incubator at 37 °C for subsequent experiments.

### RNA pulldown

Carmn probes were generated by Gzscbio (Guangzhou, China). The VSMCs were washed with PBS 2–3 times, and the cells were subsequently lysed with RIPA cell lysis buffer (Solarbio, China). Streptavidin magnetic beads (Invitrogen, SA10004) were incubated with Carmn probes for 2 h at room temperature. Total RNA was extracted, the expression of the target gene was assessed by qPCR, and the protein was quantified by SDS‒PAGE.

### Luciferase reporter assays

The potential binding sites of Carmn and its target genes were predicted using Freiburg RNA Tools. Carmn was subsequently cloned and inserted into the luciferase vector psiCHECK-2 (Gzscbio, China). To create a mutant sequence, Carmn-sv-mut, specific bases corresponding to the binding site between Carmn and the target gene were altered. Following this, 293 T cells were transfected with both the luciferase reporter vector and the mutant gene carrier. The luciferase activity for each group was then evaluated using a dual-luciferase reporter assay system (Promega, USA).

### Human aortic samples

The human aortic samples utilized in this study received approval from the Research Ethics Committee of Guangdong Provincial People’s Hospital (Ethics Approval Number, NFEC-2019–086) in accordance with the principles outlined in the Declaration of Helsinki. Each participant provided informed consent form. All AAA samples were randomly selected from patients undergoing open AAA repair. The nonaneurysmal portion of the blood vessel excised from the same patient served as the control group. Exclusion criteria for participants were aortic dissection or other inflammatory aortic diseases. After the samples were obtained, they were immediately rinsed with saline and frozen in liquid nitrogen before being stored in a − 80 °C freezer. The basic information of the patients is shown in Supplemental Table 5.

## Statistical analysis

Quantitative results are presented as mean ± standard deviation, while the fraction of elastin degradation is expressed as the median and quartile range. All experimental data were derived from independently repeated experiments. The statistical analysis and image visualization were conducted using ImageJ software (National Institutes of Health, USA) and GraphPad Prism 8.0 software (USA), respectively. Normality was tested using the Shapiro‒Wilk test. The incidence of AAA was compared using Fisher's exact test, and the survival rate was analyzed by the log-rank (Mantel‒Cox) test. A p-value of less than 0.05 was considered statistically significant.

## Results

### Single-cell RNA (scRNA) sequencing analysis reveals that CARMN is enriched in VSMCs and participates in regulating VSMC contractile activity

**Fig. 1:**
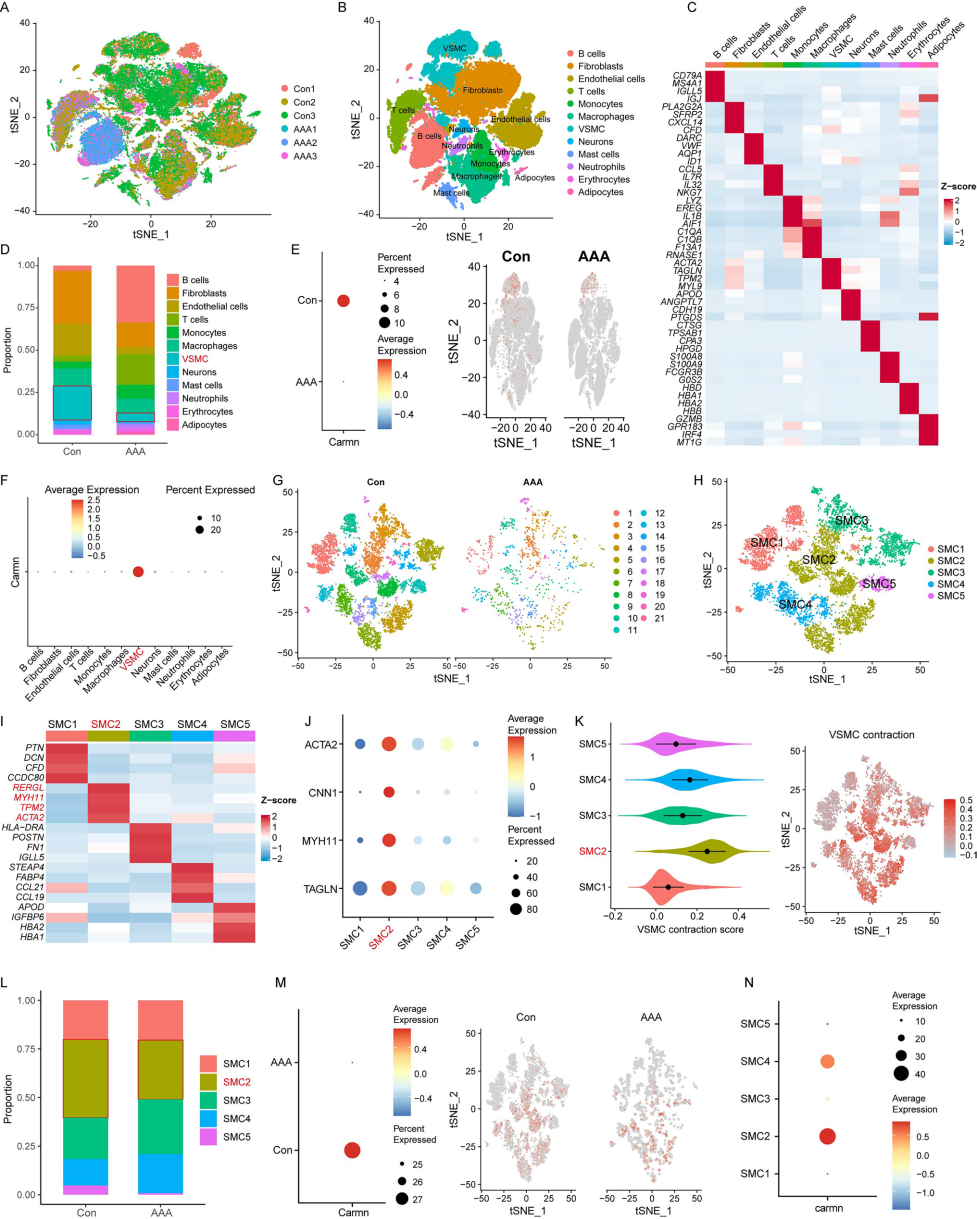
Single-cell RNA sequencing analysis reveals that CARMN is enriched in VSMCs and participates in regulating VSMC contractile activity. **A** A total of 43,208 cells in 3 normal abdominal aortic samples and 26,075 cells in 3 AAA samples were integrated and projected onto a t-SNE plot. **B** A t-SNE plot shows all cells colored according to the 12 major cell types. **C** Heatmap shows the expression of marker genes of 12 cell types. **D** Bar plots show cluster distribution within the 12 cell types in each group. **E** Dot plots show the expression of CARMN in each group (left); relative expression of CARMN in each group. Cells were projected onto a t-SNE plot (right). **F** Dot plots show the expression of CARMN in different cell types. **G** A total of 8700 VSMCs in the normal abdominal aorta group and 1,319 VSMCs in the AAA group were divided into 21 clusters and projected onto a t-SNE plot. **H** A t-SNE plot shows all VSMCs colored according to the 5 major cell types. **I** Heatmap shows the expression of marker genes of 5 VSMC types. **J** Dot plots show the expression of contraction markers (ACTA2, CNN1, MYH11, TAGLN) in 5 VSMC types. **K** Violin plots show the score of VSMC contraction in 5 VSMC types by AddModuleScore (left); relative score of VSMC contraction in VSMCs. The cells were projected onto a t-SNE plot (right). **L** Bar plots show cluster distribution within the 5 VSMC types in each group. **M** Dot plots show the expression of CARMN in VSMCs of different groups (left); relative expression of CARMN in VSMCs of different groups. Cells were projected onto a t-SNE plot (right). **N** Dot plots show the expression of CARMN in 5 VSMC types

To clarify the biological functions of CARMN in the aorta, we first performed scRNA sequencing of 3 human AAA samples and 3 normal control (Con) aorta samples. The data are available in the Gene Expression Omnibus (GEO) database (GSE226492; https://www.ncbi.nlm.nih.gov/geo/query/acc.cgi). We manually annotated the 18 clusters as 12 distinct cell populations (fibroblasts, endothelial cells, monocytes, macrophages, VSMCs, neurons, T cells, mast cells, neutrophils, erythrocytes, adipocytes, and B cells) based on the appropriate marker genes and visualized these annotations with a t-SNE plot (Fig. 1A, B). The specific cell marker expression characteristics are visualized in Fig. 1C. Compared with that in the control group, the proportion of HVSMCs was dramatically lower in the AAA group (Fig. 1D), indicating that the alteration in HVSMC content is a major cause of AAA formation. Moreover, we found that CARMN was enriched mainly in HVSMCs in the control group, while in the AAA group, CARMN expression was significantly downregulated (Fig. 1E, F).

The HVSMCs were subsequently subjected to dimension reduction and clustering (Fig. 1G), and five subpopulations (SMC1 ~ 5) were identified and visualized via t-SNE analysis (Fig. 1H). Among the 15 types of nonimmune cells, we identified five SMC or SMC-related clusters. The SMC1 cluster highly expressed extracellular-matrix-related genes such as *PTN*, *DCN* and *CFD*. SMC2 cluster highly expressed contraction-related genes such as *MYH11*, *ACTA2*. SMC3 cluster highly expressed extracellular-matrix-related and immune-related genes such as *POSTN*, *HLA-DRA*, *FN1* and *IGLL5*. SMC4 cluster highly expressed fatty acid metabolism related and immune-related genes such as *CCL21*, *CCL19* and *FABP4*. SMC4 cluster highly expressed fatty acid metabolism related and immune-related genes such as *CCL21*, *CCL19* and *FABP4*. SMC5 highly expressed genes related to hemoglobin such as *HBA1*, *HBA2* (Fig. 1I, J). The VSMC contractile gene set was obtained from the GSEA database to evaluate the contractile activity of the five subpopulations using the Addmodule method, and we found that the SMC2 subpopulation possessed the highest contractile activity (Fig. 1K). The AAA group had a lower proportion of SMC2 (Fig. 1L), indicating that the decrease in the contractile SMC2 subpopulation was related to AAA formation. Interestingly, CARMN was highly abundant in SMC2 cells in the control group, and its expression was downregulated in parallel with the reduction in SMC2 expression in the AAA group (Fig. 1M, N), suggesting that CARMN may participate in regulating HVSMC contractile activity during the progression of AAA.

In mice, we obtained similar results through analysis of the scRNA sequencing dataset (GSE152583), which includes AAA samples from mice treated with PPE for 7 days (AAA_7d) and 14 days (AAA_14d) and normal samples from sham-operated mice (Sham). Based on the known marker genes, ten cell clusters (VSMCs, fibroblasts, macrophages, T cells, monocytes, erythrocytes, endothelial cells, B cells, adipocytes, dendritic cells) were identified (Supplemental Figure IA-B). CARMN was enriched in MVSMCs and downregulated in AAA samples (Supplemental Figure IC). Five VSMC subpopulations were defined, and SMC1 was recognized as contractile VSMCs with the highest VSMC marker expression and contraction score (Supplemental Figure ID-G). CARMN was highly expressed in the subpopulation SMC1 (Supplemental Figure IH), which also indicated that CARMN might maintain the contractile function of VSMCs.

Taken together, these results revealed the role of CARMN in regulating VSMC contractile activity in the progression of AAA.

### Bulk RNA sequencing analysis reveals that CARMN expression is downregulated in mouse AAA tissue and associated with VSMC function

**Fig. 2:**
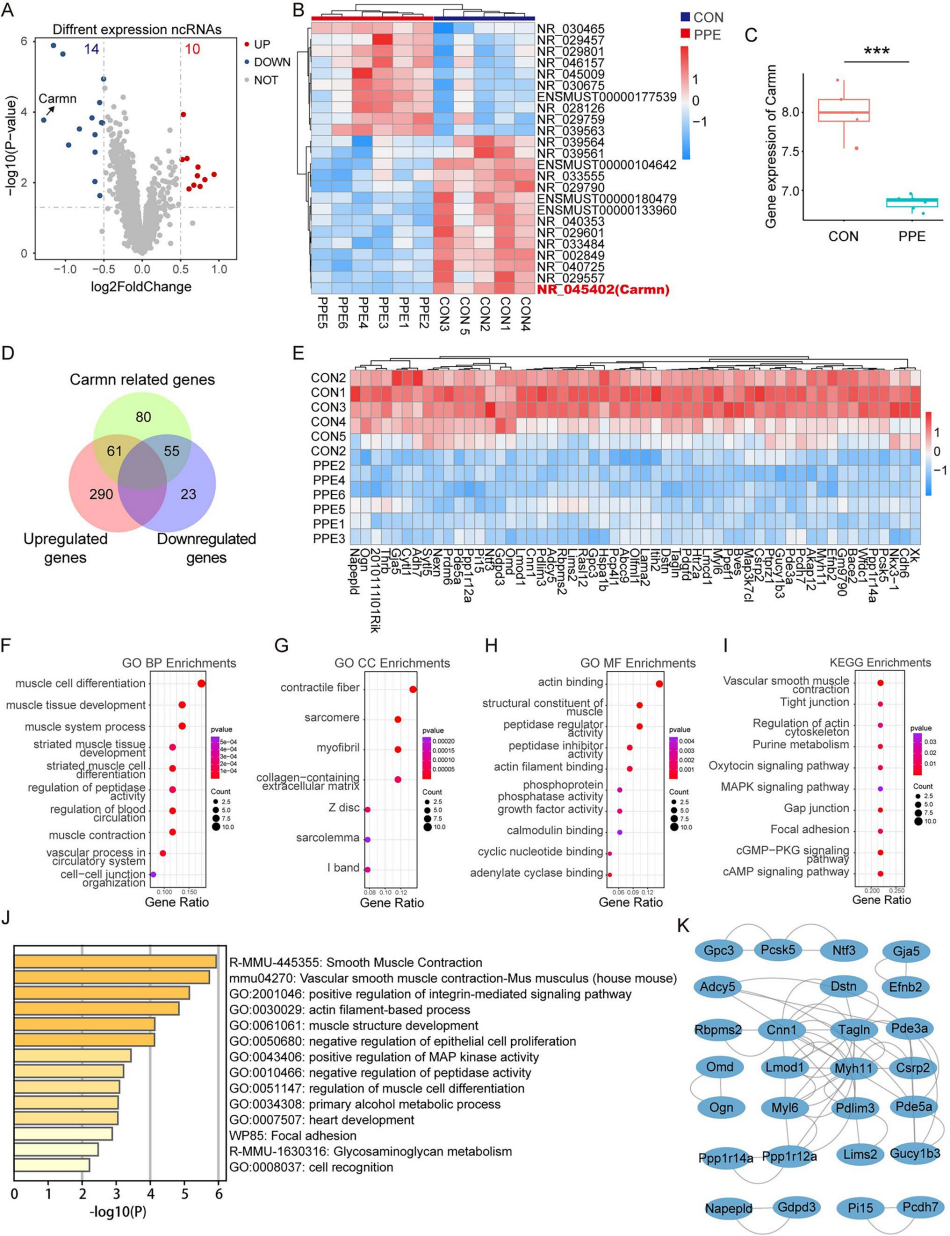
Bulk RNA sequencing analysis reveals that CARMN expression is downregulated in mouse AAA tissue and associated with VSMC function. **A** Volcano plot shows the differential expression of ncRNAs between PPE-induced AAA samples and control samples. **B** Heatmap shows the differential expression of ncRNAs in each group. **C** The differential expression of Carmn between AAA samples and control samples (***p = 0.00091). **D** Venn diagram shows the intersection of Carmn-related mRNAs, upregulated mRNAs and downregulated mRNAs in AAA samples. **E** Heatmap shows the differential expression of mRNAs coexpressed with Carmn in each group. **F**–**H** GO analysis of mRNAs coexpressed with Carmn. **I** KEGG analysis of mRNAs coexpressed with Carmn. **J** Metascape analysis of mRNAs coexpressed with Carmn. **K** String analysis shows the PPI network of mRNAs coexpressed with Carmn

To further investigate the expression and functional characteristics of CARMN during AAA formation, we analyzed the transcriptional profiling dataset (GSE226736) of 7-day PPE-induced mouse AAA samples (PPE, n = 6) and untreated normal control aortic samples (CON, n = 5). The raw data were standardized and reannotated using the rma method. The expression levels of ncRNAs and mRNAs were basically consistent across all the samples (Supplemental Figure IIA and IIC), suggesting that there is no dimensional difference or batch effect that may disrupt the downstream analysis. The PCA results revealed the characteristic distinctions of ncRNA and mRNA expression data between PPE-induced aortas and the controls (Supplemental Figure IIB and IID). According to the standard of adjusted *p* values < 0.05 and |log2FC|> 1.5, the DEGs were explored, and the dysregulated ncRNAs and mRNAs that may be associated with AAA formation were visualized by volcano plots and heatmaps. A total of 14 downregulated and 10 upregulated ncRNAs were identified, and CARMN exhibited the highest fold change among the downregulated ncRNAs (Fig. 2A–C). We also identified 351 upregulated and 78 downregulated mRNAs (Supplemental Figure IIIA-B). GO and KEGG analyses of these differentially expressed mRNAs showed they were mainly associated with immunity, inflammation, cytokine and chemokine biological functions and pathways (Supplemental Figure IIIC-F). The changes in these functions and pathways induced by the dysregulation of genes were closely related to the formation of AAA.

As lncRNAs often function by regulating mRNAs, we then performed Pearson coexpression analysis to identify the significantly positively coexpressed mRNAs with CARMN. We identified 55 downregulated mRNAs that might be regulated by CARMN in PPE samples (Fig. 2D, E) and further performed GO and KEGG analyses. The terms were mainly related to cell differentiation, cell adhesion biological functions and VSMC pathways (VSMC muscle contraction) (Fig. 2F–I). The above findings suggest that CARMN is a major regulator involved in VSMC pathways during AAA formation. We further explored the association between CARMN-regulated mRNAs and VSMC pathways. The functionally enriched pathway of CARMN-regulated mRNAs provided by the Metascape database (http://metascape.org/), a tool for gene function annotation, is vascular smooth muscle contraction (Fig. 2J). The String database provided the protein‒protein interaction (PPI) relationship of the proteins expressed by the upregulated and downregulated mRNAs. Consistently, these proteins are closely connected and affect muscle fibers and the cytoplasmic matrix (contractile fiber, sarcomere, contractile actin filament bundle, and cell-substrate junction) (Fig. 2H). These findings suggest that CARMN is closely related to VSMC function.

We also analyzed another published profiling dataset (GSE197748) of infrarenal aortas from both male and female mice treated with PPE (aneurysm group) or saline (control group) for 7 days to investigate CARMN expression in AAA in both sexes and obtained consistent results. After standardization as described above, we found a significant difference in gene expression between the two groups (Supplemental Figure IVA-D). Upregulated and downregulated ncRNAs were identified, and CARMN was also obviously decreased in AAA (Supplemental Figure IVE-G). GO and KEGG analyses of the differentially expressed mRNAs showed they were related to immunity, inflammation, cytokines, and chemokines (Supplemental Figure V). We then obtained 30 downregulated mRNAs that were coexpressed with CARMN by performing Pearson coexpression analysis (Supplemental Figure VIA). Similarly, GO and KEGG analyses of the 30 mRNAs mainly identified molecular functions related to cell differentiation, metabolism and chemokines and VSMC pathways (vascular smooth muscle contraction) (Supplemental Figure VIB-E). Functional enrichment analysis conducted by the Metascape database also showed that the top enriched functional pathway of the 30 mRNAs was the VSMC pathway (Supplemental Figure VIF). The PPI network applied by the GeneMANIA database suggested that the functions of the 30 proteins were related to muscle fibers and cytoplasmic matrix (such as contract fiber, sarcomere, contract action filling bundle, cell substrate junction, etc.) (Supplemental Figure VIG).

These findings from bulk RNA sequencing analysis demonstrate that CARMN expression was substantially reduced in mouse AAA and associated with VSMC function.

### CARMN expression is decreased in mouse and human AAA tissues

**Fig. 3:**
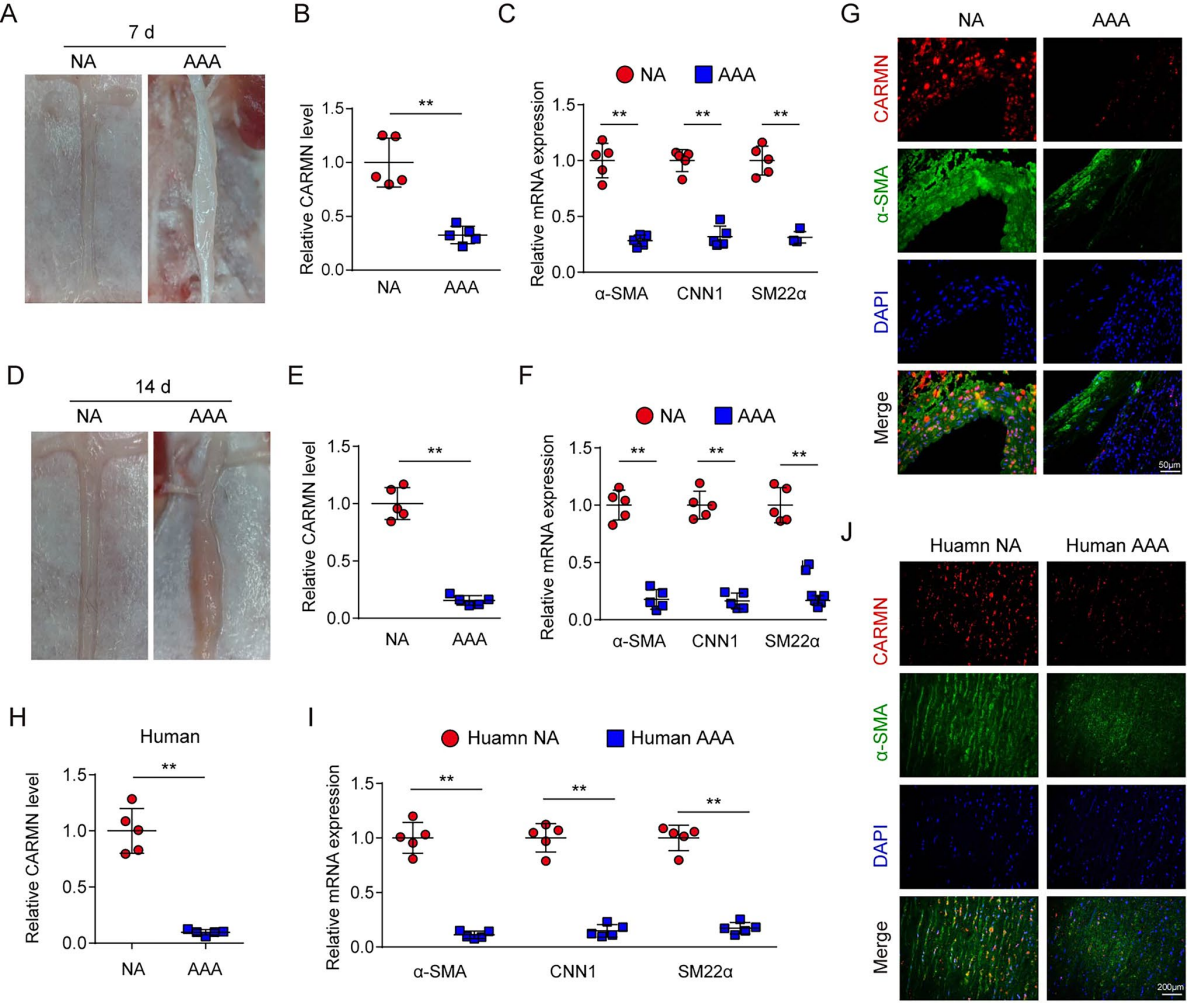
CARMN is decreased in mouse and human AAA tissues. **A** Representative photographs show mouse abdominal aortas treated with PPE (AAA) or saline (NA) for 7 days. **B** Relative CARMN levels detected by qPCR in NA or AAA (**p < 0.01, n = 5 in each group). **C** Relative mRNA levels of α-SMA, CNN1 and SM22α detected by qPCR in NA or AAA (**p < 0.01, n = 5 in each group). **D** Representative photographs show mouse abdominal aortas treated with PPE (AAA) or saline (NA) for 14 days. **E** Relative CARMN levels detected by qPCR in NA or AAA (**p < 0.01, n = 5 in each group). **F** Relative mRNA levels of α-SMA, CNN1 and SM22α detected by qPCR in NA or AAA (**p < 0.01, n = 5 in each group). **G** FISH of CARMN (red) and immunofluorescence staining of α-SMA (green) and DAPI (green) in mouse abdominal aortas treated with either PPE (AAA) or saline (NA) for 14 days. **H** Relative CARMN levels detected by qPCR in human NA or AAA (**p < 0.01, n = 5 in each group). **I** Relative mRNA levels of α-SMA, CNN1 and SM22α detected by qPCR in human NA or AAA (**p < 0.01, n = 5 in each group)

To determine the expression characteristics of CARMN, we first tested its expression in different tissues, including aortas, hearts, lungs, livers, brains and skeletal muscles. qPCR revealed that CARMN was highly expressed in the aorta (Supplemental Figure VII). To confirm the abnormal expression of CARMN, we established mouse AAA models by external stimulation with PPE, and the controls were treated with saline. We observed significant arterial dilation in the infrarenal abdominal aorta 7 days after PPE stimulation (Fig. 3A). qPCR showed that CARMN expression was decreased in PPE-induced AAA tissues, consistent with the downregulation of known VSMC contractile marker genes, including α-smooth muscle actin (α-SMA), calponin 1 (CNN1) and smooth muscle 22α (SM22α) (Fig. 3B, C). Similarly, 14 days after PPE stimulation, CARMN and VSMC contractile marker genes showed a more significant decrease in AAA tissues (Fig. 3E, F). To obtain more information on the localization of CARMN in the aorta, we performed in situ hybridization (ISH) staining on AAA tissue 14 days after PPE stimulation and on the control tissue. CARMN was found to be expressed mainly in the medial VSMC layer through colocalization of CARMN and α-SMA and was downregulated in AAA aortic walls (Fig. 3G).

To establish the clinical relevance of CARMN and AAA formation, we further collected AAA samples from patients who underwent open AAA repair surgery. The nonaneurysmal portion of the blood vessel excised from the same patient served as the control group. The downregulation of CARMN and VSMC contractile marker genes was also confirmed in clinical human AAA samples by qPCR (Fig. 3H). CARMN was also found to be localized in the medial VSMC layer of the aorta (Fig. 3J).

These results also showed that CARMN expression is downregulated in mouse and human AAA tissues.

### CARMN knockdown exacerbates abdominal aortic aneurysm formation in mice

**Fig. 4:**
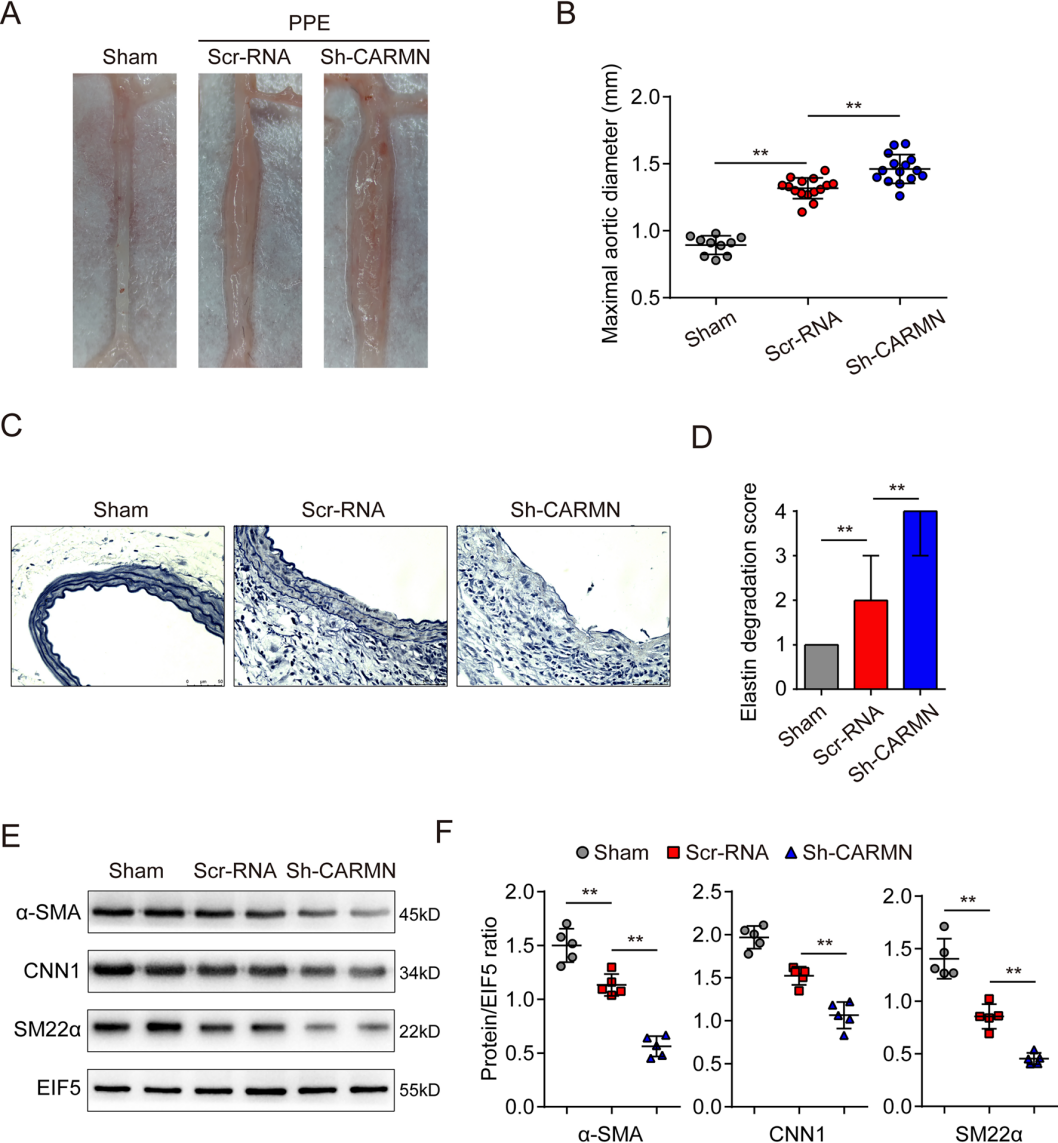
Knockdown of CARMN aggravates AAA formation in mice. **A** Representative photographs show the abdominal aortas from the sham group mice, PPE-treated Scr-RNA group mice and PPE-treated Sh-CARMN group mice. **B** Maximal abdominal aortic diameters of each group of mice (**p < 0.01, n = 10 in the sham group, n = 15 in the Scr-RNA group and Sh-CARMN group). **C** and **D** Representative staining with elastin and the elastin degradation score of the abdominal aortas from Ang II–infused mice. Photographs were taken at the location where the most severe elastin degradation occurred (scale bars = 50 µm, **p < 0.01; n = 8 in sham group, n = 15 in Scr-RNA group and Sh-CARMN group). **E** and **F** Protein levels of α-SMA, CNN1 and SM22α detected by western blots in each group. EIF5 was used as a loading control (**p < 0.01, n = 5 in each group)

To investigate the function of CARMN in AAA, we used an adeno-associated virus serotype 9 (AAV9) carrier against CARMN (Sh-CARMN) to inhibit CARMN expression in vivo. Scrambled RNAs were used as relevant controls (Scr-RNA). The temporal process of viral transfection and model establishment is shown in Supplemental Figure VIIIA. Viral infection was performed 30 days before mouse model establishment. Immunofluorescence staining for green fluorescent protein (GFP) indicated effective transfection of VSMCs (stained with SM22α) into the aortic wall after 30 days of infection (Supplemental Figure VIIIB). The knockdown effect was then confirmed by qPCR analysis (Supplemental Figure VIIIC). Then, AAA was induced in CARMN knockdown or control mice through PPE stimulation. Wild-type mice that underwent open surgeries and were stimulated with saline under similar conditions were regarded as the sham group. Arteries were harvested 14 days after surgery, and varying degrees of aneurysm formation were observed in the PPE-stimulated Scr-RNA group and Sh-CAMRN group, while no aortic expansion was found in the sham group. Upon PPE stimulation, knockdown of CARMN significantly exacerbated AAA development, as shown by the expanded diameters (Fig. 4A and B). Elastic fiber staining showed that knockdown of CARMN also deteriorated the degradation of elastic fibers (Fig. 4C and D). Moreover, PPE stimulation decreased the mRNA and protein expression of VSMC contractile markers, including α-SMA, CNN1 and SM22α, and CARMN knockdown significantly inhibited the expression of VSMC contractile markers (Supplemental Figure VIIID, Fig. 4E and F), suggesting that CARMN knockdown induces VSMC phenotypic transformation to the secretory phenotype.

These results indicate that CARMN knockdown exacerbates mouse AAA and induces VSMC phenotypic transformation to the secretory phenotype.

### CARMN overexpression attenuates AAA formation in mice

**Fig. 5:**
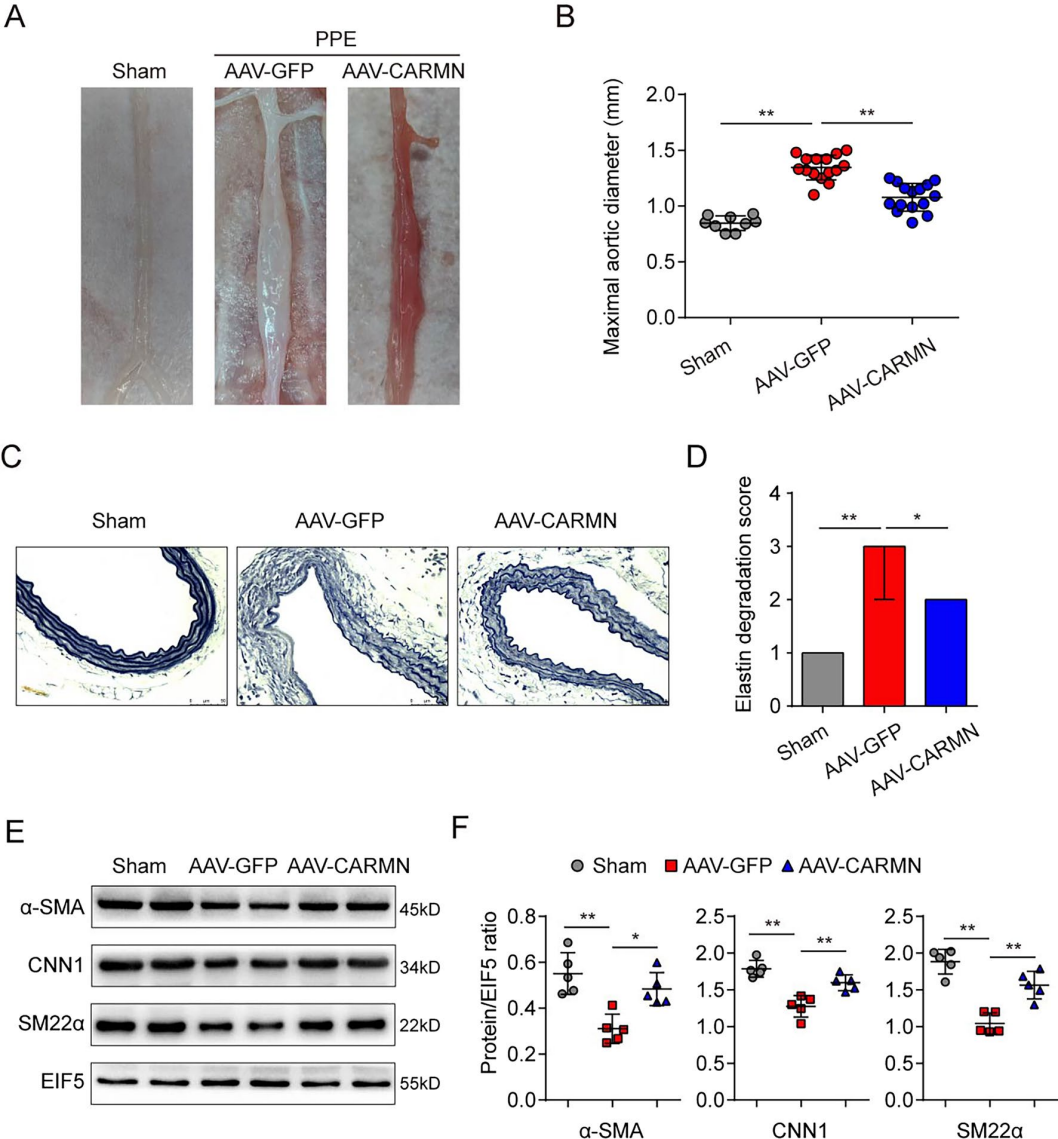
CARMN overexpression attenuates AAA formation in mice. **A** Representative photographs show the abdominal aortas from the sham group mice, PPE-treated AAV-GFP group mice and PPE-treated AAV-CARMN group mice. **B** Maximal abdominal aortic diameters of each group of mice (**p < 0.01, n = 9 in the sham group, n = 15 in the AAV-GFP group and AAV-CARMN group). **C** and **D** Representative staining with elastin and the elastin degradation score of the abdominal aortas from Ang II–infused mice. Photographs were taken at the location where the most severe elastin degradation occurred (scale bars = 50 µm, **p < 0.01, *p < 0.05; n = 9 in the sham group, n = 15 in the AAV-GFP group and AAV-CARMN group). **E** and **F** Protein levels of α-SMA, CNN1 and SM22α detected by western blots in each group. EIF5 was used as a loading control (**p < 0.01, *p < 0.05, n = 5 in each group)

To further determine whether CARMN influences AAA formation in vivo, mice were infected with either CARMN-overexpressing AAV9 (AAV-CARMN) or control AAV9 (AAV-GFP) to investigate the effect of CARMN overexpression on AAA formation. After 30 days of infection, mouse aortas were isolated, and immunofluorescence staining and qPCR confirmed that the viruses were successfully transfected into VSMCs and increased the expression of CARMN (Supplemental Figure IXA-B). PPE stimulation induced AAA formation in the AAV-GFP and AAV-CARMN groups. However, we observed that PPE-induced AAA was significantly restored by CARMN overexpression (Fig. 5A and B). CARMN overexpression also prominently alleviated the elastic fiber degradation caused by PPE (Fig. 5C and D). The results of qPCR and western blotting demonstrated that PPE stimulation induced the downregulation of VSMC contractile marker genes, including α-SMA, CNN1 and SM22α, while CARMN overexpression reversed this effect, suggesting that CARMN contributes to maintaining the contractile phenotype of VSMCs (Supplemental Figure IXC, Fig. 5E and F).

These results further verify the role of CARMN in AAA formation and VSMC phenotypic transformation.

### CARMN interacts with SRF to modulate the smooth muscle cell phenotype

**Fig. 6:**
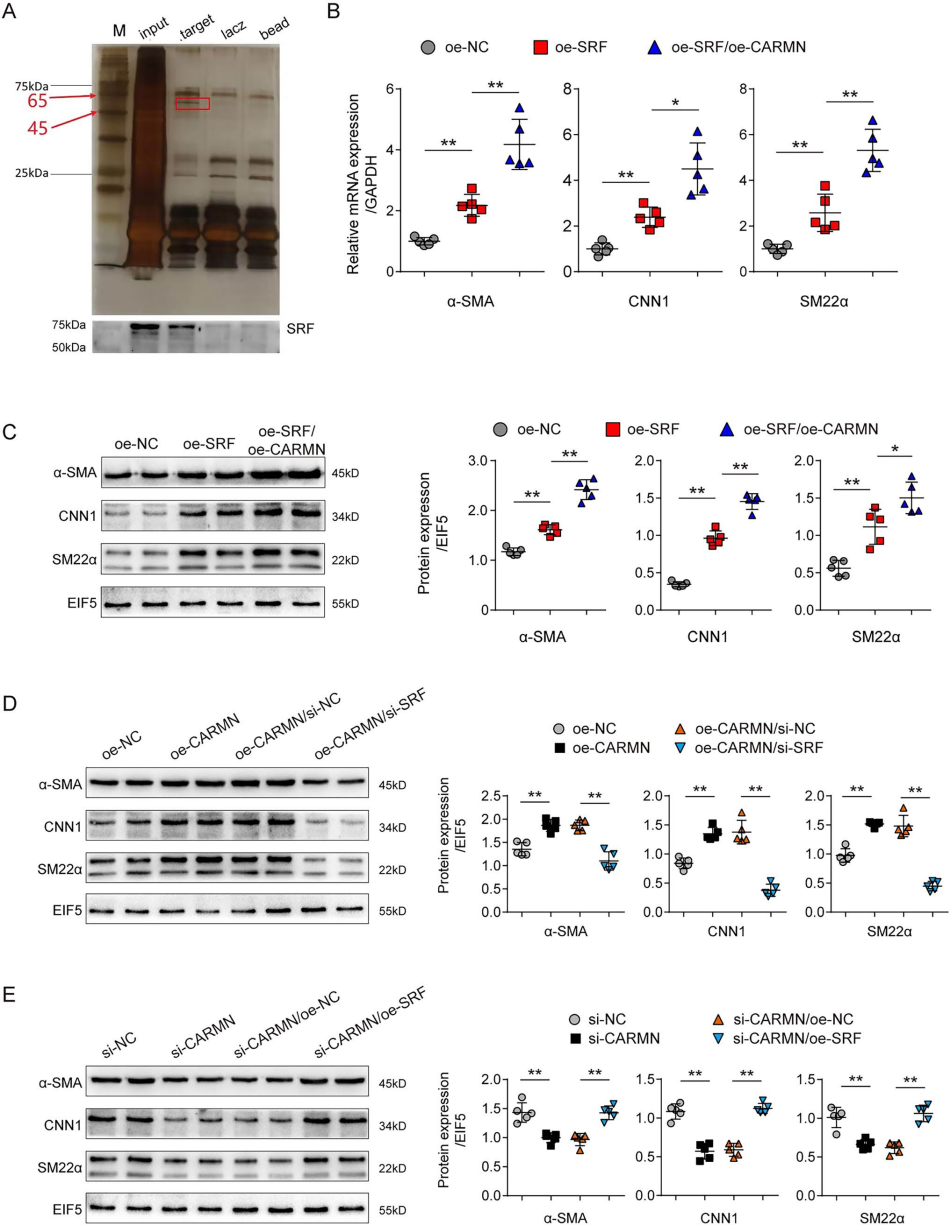
CARMN binds to SRF to modulate the smooth muscle cell phenotype. **A** Protein immunoprecipitated by the probe against CARMN and the control. Bottom, SRF protein was detected by western blotting. **B** Relative mRNA levels of α-SMA, CNN1 and SM22α detected by qPCR in mouse primary VSMCs treated with empty vector (oe-NC), SRF overexpression plasmids (oe-SRF), or SRF overexpression plasmids and CARMN overexpression plasmids (oe-SRF/oe-CARMN) (**p < 0.01, *p < 0.05, n = 5 in each group). **C** Protein levels of α-SMA, CNN1 and SM22α detected by western blots in each group indicated in (**B**). EIF5 was used as a loading control (**p < 0.01, *p < 0.05, n = 5 in each group). **D** Protein levels of α-SMA, CNN1 and SM22α detected by western blots in mouse primary VSMCs treated with empty vector (oe-NC), CARMN overexpression plasmids (oe-CARMN), CARMN overexpression plasmids and control siRNAs (oe-CARMN/si-NC), or CARMN overexpression plasmids and SRF siRNAs (oe-CARMN/si-SRF) (**p < 0.01, n = 5 in each group). **E** Protein levels of α-SMA, CNN1 and SM22α detected by western blots in mouse primary VSMCs treated with control siRNAs (si-NC), CARMN siRNAs (si-CARMN), CARMN siRNAs and empty vector (si-CARMN/oe-NC), or CARMN siRNAs and SRF overexpression plasmids (si-CARMN/oe-SRF) (**p < 0.01, n = 5 in each group)

To explore the downstream targets of CARMN during AAA formation, we performed an RNA pulldown assay followed by mass spectrometry analysis to identify potential CARMN-interacting proteins (Fig. 6A). Supplemental Table 6 shows the proteins that may bind to CARMN identified by mass spectrometry analysis. Among these proteins, serum response factor (SRF), a transcription factor associated with the transcription of VSMC contractile marker genes [33], has attracted our attention. For determination of the consequence of the binding of CARMN and SRF, primary VSMCs from mouse aortas were isolated and treated with SRF overexpression vectors (oe-SRF) or controls (oe-NC). SRF overexpression promoted the mRNA and protein expression of VSMC contractile markers. On this basis, overexpression of CARMN enhanced the promotional effect of SRF (Fig. 6B and C). To further validate this finding, we overexpressed CARMN in primary VSMCs. As expected, CARMN overexpression promoted the expression of VSMC contractile markers. Knocking down SRF with a small interfering RNA against SRF (si-SRF) significantly blocked the effect of CARMN (Fig. 6D). In contrast, SRF overexpression reversed the suppressive effect of CARMN knockdown on VSMC contractile phenotypic transformation (Fig. 6E). These results revealed that CARMN may exert its function by interacting with SRF.

**Fig. 7:**
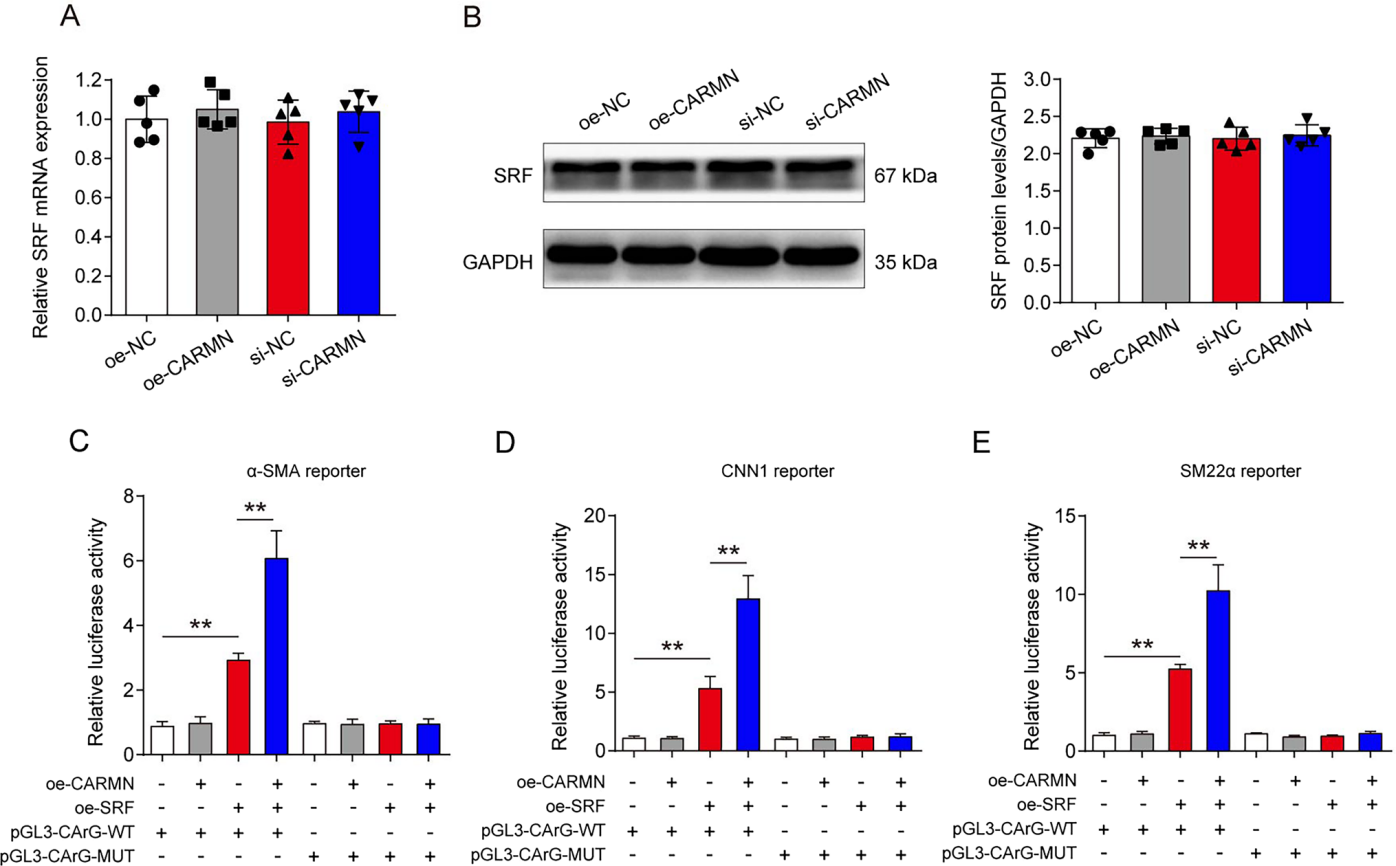
CARMN enhances the promotional effect of SRF on VSMC marker gene transcription. **A** Relative mRNA levels of SRF in mouse VSMCs after treatment with oe-NC, oe-CARMN, si-NC or si-CARMN (n = 5 in each group). **B** SRF protein levels in mouse VSMCs after treatment with oe-NC, oe-CARMN, si-NC or si-CARMN (n = 5 in each group). **C**–**E** Relative luciferase activity of wild-type (WT) or mutated (MUT) CArG box VSMC marker genes after treatment with or without oe-CARMN and oe-SRF (**p < 0.01, n = 3 in each group)

Next, we investigated the detailed effect of CARMN on SRF. Interestingly, CARMN overexpression or knockdown did not affect SRF mRNA or protein levels (Fig. 7A and B). As a transcription factor, SRF has been reported to regulate the VSMC phenotype by binding to CArG elements and transcriptionally activating VSMC contractile genes [34]. The wild-type or mutated CArG promoters (pGL3-CArG-WT or pGL3-CArG-MUT) of α-SMA, CNN1 and SM22α were constructed. We transfected 293 T cells with CARMN overexpression plasmids, SRF overexpression plasmids, and pGL3-CArG-WT or pGL3-CArG-MUT and performed luciferase reporter gene assays. We observed that overexpression of CARMN alone did not alter the expression of VSMC markers, while SRF overexpression increased the expression of these markers. On the basis of the SRF overexpression data, CARMN could significantly enhance the expression of VSMC contractile markers. However, SRF and CARMN overexpression did not alter the expression of VSMC markers when the CArG gene was mutated (Fig. 7C–E), which suggested that CARMN promoted the transcriptional effect of SRF on VSMC contractile marker genes.

Together, these results suggest that CARMN could interact with SRF and enhance its promotional effect on VSMC marker gene transcription.

### The transcription of *Carmn* is enhanced by NRF2 binding to its promoter region

**Fig. 8:**
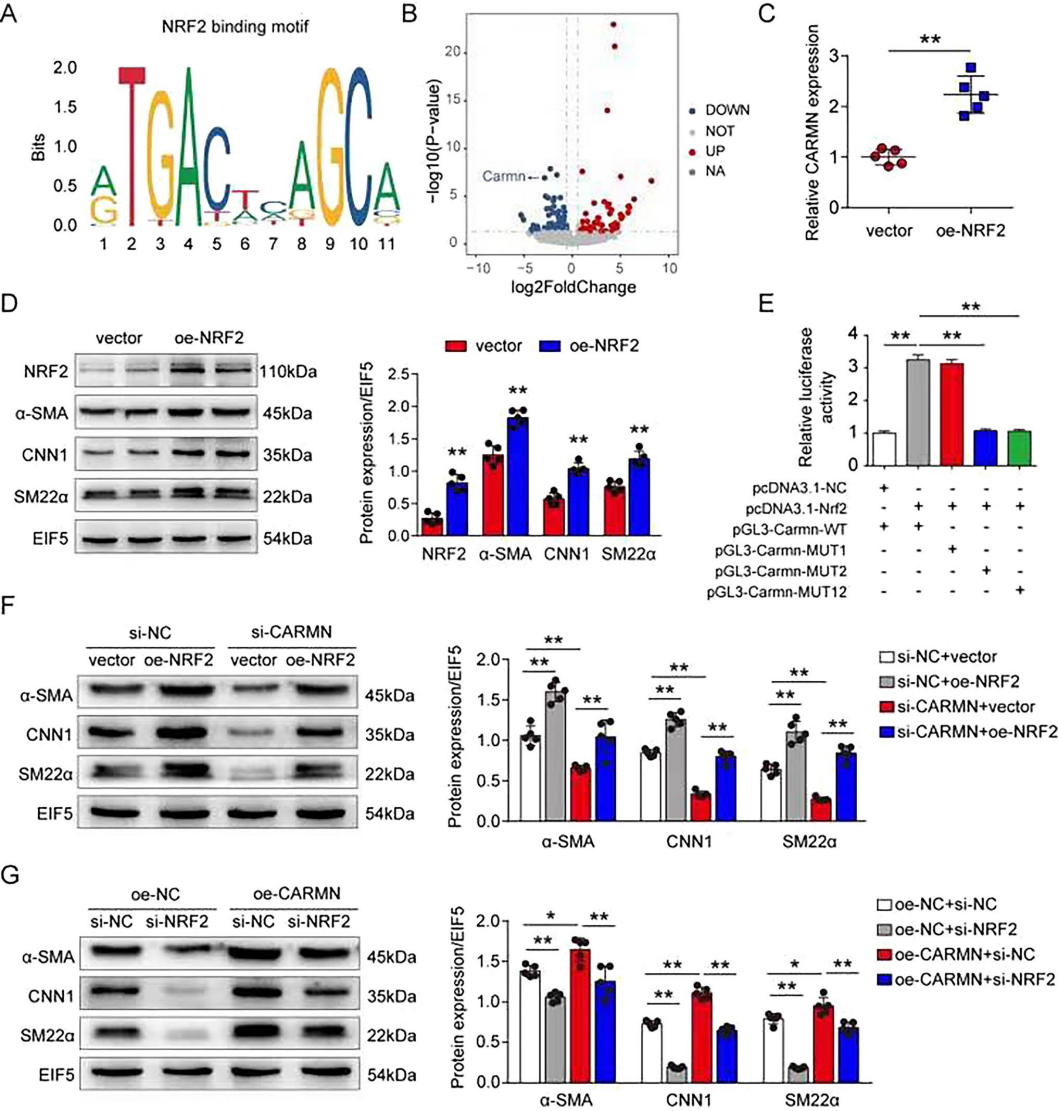
The transcription of Carmn is enhanced by NRF2 binding to its promoter region. **A** The predicted NRF2 binding motif. **B** Volcano plot of lncRNAs identified by genome-wide gene expression analysis of aortas from VSMC-specific NRF2 knockout mice and control mice (n = 3 in each group). **C** Relative CARMN levels in mouse primary VSMCs treated with vector or NRF2 overexpression plasmids (oe-NRF2) (**p < 0.01, n = 5 in each group). **D** Protein levels of Nrf2, α-SMA, CNN1 and SM22α detected by western blots in mouse primary VSMCs treated with vector or NRF2 overexpression plasmids (oe-NRF2) (**p < 0.01, n = 5 in each group). **E** Relative luciferase activity of wild-type (WT) or promoter-mutated (MUT1, MUT2 and MUT12) Carmn after treatment with or without NRF overexpression (**p < 0.01, n = 3 in each group). **F** Protein levels of α-SMA, CNN1 and SM22α detected by western blots in mouse primary VSMCs treated with si-NC or si-CARMN and then treated with empty vector or oe-NRF2 (**p < 0.01, n = 3 in each group). **G** Protein levels of α-SMA, CNN1 and SM22α detected by western blots in mouse primary VSMCs treated with oe-NC or oe-CARMN and then treated with si-NC or si-NRF2 (**p < 0.01, *p < 0.05, n = 3 in each group)

We then aimed to identify the transcription factor responsible for *Carmn* expression. To predict the probable binding sites on *Carmn*, we utilized the Jaspar database (http://jaspar.binf.ku.dk/) with default settings, and interestingly, we found that NRF2 may bind to the promoter region of *Carmn* (Fig. 8A). Genome-wide gene expression analysis revealed that CARMN was downregulated after VSMC-specific knockout of NRF2 (Fig. 8B). NRF2 increased CARMN levels, as well as downstream VSMC marker genes, as shown by qPCR and western blot assays (Fig. 8C and D). To confirm the binding between NRF2 and the *Carmn* gene, we predicted two binding sites that might be bound by NRF2. Mutant *Carmn* promoter templates were constructed according to the predicted binding sites 1 and 2 and both 1 and 2 (pGL3-Carmn-MUT1, pGL3-Carmn-MUT2 and pGL3-Carmn-MUT12). The wild-type *Carmn* promoter templates (pGL3-Carmn-WT) were regarded as the control group. The NRF2 templates (pcDNA3.1-NRF2) and the corresponding normal control templates (pcDNA3.1-NC) were connected to pcDNA3.1( +) vectors. We transfected pcDNA3.1-NRF2/pcDNA3.1-NC or pGL3-Carmn-MUT1/pGL3-Carmn-MUT2/pGL3-Carmn-MUT12 into 293 T cells and performed luciferase reporter gene assays to detect the regulatory effect of NRF2 on *Carmn*. The expression of luciferase was restricted when binding site 2 or both sites 1 and 2 were mutated (Fig. 8E), suggesting that NRF2 can bind to site 2 in the *Carmn* promoter region. Furthermore, western blot analyses revealed that the decrease in VSMC markers induced by CARMN inhibition in VSMCs could be reversed by NRF2 overexpression, while the increase in VSMC markers induced by CARMN overexpression could be restricted by NRF2 knockdown (Fig. 8F and G).

These findings demonstrated that the transcription of *Carmn* is enhanced by NRF2 binding to its promoter region.

## Discussion

In our study, we identified a VSMC-enriched lncRNA CARMN that was significantly downregulated in human and mouse AAA specimens, correlating with VSMC contractile function. Through gain- and loss-of-function experiments, we established CARMN’s protective influence in AAA development. On a mechanistic level, CARMN was found to interact with SRF’s role in boosting the transcription of VSMC contractile marker genes, thus preserving the contractile phenotype of VSMCs. In addition, the transcription of CARMN can be positively regulated by binding of NRF2 to its promoter region, thus promoting VSMC phenotypic transformation to the contractile phenotype.

LncRNAs, a relatively recent discovery in the realm of RNA, have been recognized for their regulatory roles across various physiological and pathological processes, including disease progression. Numerous studies have indicated the capability of lncRNAs to modulate the phenotypic transformation of VSMC [35–37] and their impact on cardiovascular diseases, including AAA [19–21]. However, the lack of VSMC-specific expression in many of these lncRNAs may hinder their therapeutic potential in AAA management. Cardiovascular diseases are main killer of people all over the world [38–41]. Therefore, to enhance the prospects of clinical application, our focus shifted to investigating an lncRNA predominantly expressed in VSMCs as a potential therapeutic target for AAA. CARMN, a predominant and conserved lncRNA in SMCs, was initially identified as crucial in cardiac development, maintaining differentiated cardiac states in pathological heart remodeling [42]. Its expression, notably diminished in atherosclerosis and diseases involving VSMC phenotypic modulation [22, 24], also decreases upon stimulation by factors like PDGF-BB or TGF-β1 [24, 25]. CARMN works by physically binding to Myocardin, a key transcriptional cofactor, thus promoting its activity to preserve the VSMC contractile state [22]. Additionally, CARMN interacts with serum response factor (SRF) to regulate VSMC plasticity, significantly impacting the progression of atherosclerosis [25]. It is also crucial for gastrointestinal (GI) tract SMCs, as its absence leads to phenotypic shifts and impacts contractile gene expression, vital for GI motility [43]. Furthermore, CARMN’s downregulation in AAA, possibly mediated by inflammatory macrophages, correlates with a loss of the SMC contractile state [44]. CCL21 and CCL19, chemokines involved in vascular inflammation and atherosclerosis, might be modulated by CARMN to regulate immune cell behavior affecting VSMCs. Similarly, CARMN might influence SMC functions by regulating metabolic pathways or inflammatory responses associated with FABP4, a protein active in lipid metabolism and inflammation, particularly in atherosclerosis and AAA.

Single-cell sequencing technology has been instrumental in our research for elucidating the phenotypic transitions of SMCs in AAA development. This study distinguished various cell types through gene expression patterns in single-cell data, highlighting significant shifts in SMC subtypes within AAA. Notably, a marked decrease in contractile SMCs was observed. Our experiments, both in vivo and in vitro, indicated that CARMN plays a role in inhibiting AAA development by preserving the contractile homeostasis of SMCs. This aligns with previous findings that VSMCs’, reduced contractility and transition to a synthetic phenotype, involving extracellular matrix remodeling and inflammation, which promoting the progression of AAA [45]. Meanwhile, our result of single-cell sequencing analysis indicated that VSMCs might regulate vascular homeostasis through its own functional homeostasis and phenotypic transitions. In addition, relevant study revealed that the loss of Bestrophin3 inhibited the phenotypic regulation of contractile SMCs towards fibroblastic cells through single-cell sequencing, resulting in a reduction of vascular wall scaffold proteins and extracellular matrix, ultimately leading to the imbalance of vascular homeostasis [46]. Lineage tracing combined with single-cell sequencing has further demonstrated the significant impact of SMCs on atherosclerosis progression [47]. The development of single-cell multi-omics technologies and single-cell spatial transcriptomics further explored the composition, interactions, and functions of cells in complex tissues and organs [48]. In the future, it is imperative to integrate the latest single-cell sequencing technologies to find out the connection between vascular functional homeostasis and VSMC phenotypes, which represents an effective research strategy that can contribute to the identification of promising therapeutic targets. In this study, we further verified a reduction of CARMN in human and mouse AAA tissues, suggesting its potential role in AAA formation.

VSMCs, the major component of the aortic wall, maintain the normal structure and physiological function of the vasculature [7]. The highly plastic character allows VSMCs to transition between contractile and secretory phenotypes in response to environmental stimuli and extracellular signals [49]. A previous study revealed that the phenotypic transformation of VSMCs to the secretory phenotype likely occurred before AAA formation according to the elastase perfusion model [50]. Regulating VSMC phenotypic transformation may be a potential method for preventing and treating AAA. MiR-23b was found to be downregulated in AAA and can stabilize the VSMC contractile phenotype and protect against AAA formation by inhibiting the expression of the transcription factor forkhead box O4 (FoxO4) [51]. BAF60c has been reported to maintain the VSMC contractile phenotype by serving as a coactivator of serum response factor (SRF), and its loss exacerbates AAA development [52]. To understand the specific role of CARMN in AAA and VSMC phenotypic transformation, we chose a classical murine model established by the external application of PPE to imitate the pathological characteristics of human AAA. Functional experiments indicated that knockdown of CARMN aggravated AAA formation, while overexpression of CARMN had the opposite effect. Previous studies have identified α-SMA, CNN1 and SM22α as contractile VSMC marker genes that maintain VSMC function and prevent vascular diseases, including AAA. Herein, we observed significant changes in the expression of VSMC marker genes in PPE-induced AAA tissues after CARMN knockdown or overexpression. These findings suggested that CARMN affects AAA formation by regulating VSMC phenotypic transition.

To explore the potential pathological mechanisms of CARMN during AAA formation, we performed RNA pulldown followed by mass spectrometry. These results indicated that CARMN may interact with SRF. SRF is a widely expressed transcription factor involved in muscle differentiation, cellular growth and motility [33]. Developmental analysis demonstrated that the highest levels of SRF gene expression occur in differentiating cardiac, skeletal, and smooth muscle cell lineages [53]. As a transcription factor, SRF binds to the CArG (CC[A/T]6GG) box of VSMC contractile marker gene promoters, together with its cofactor Myocardin, to activate the transcription of α-SMA, CNN1, SM22, MYH11, etc., and maintain the contractile phenotype of VSMCs. Our results showed that the SRF-induced upregulation of VSMC contractile marker genes can be enhanced by the overexpression of CARMN. In addition, interfering with SRF reversed the modulatory effect of CARMN on the VSMC phenotype. Moreover, we confirmed that the effect of CARMN on SRF occurs not by increasing the expression of SRF but by enhancing its own role as a transcription factor to promote downstream VSMC contractile marker gene transcription. Our data echo those of previous studies showing that CARMN plays a crucial role in regulating SRF binding to promoter regions of target VSMC genes [25]. LncRNAs engage in epigenetic regulation by modulating chromatin structure and dynamics, often interacting with chromatin-modifying enzymes to influence gene accessibility [54–56]. Additionally, lncRNAs play a significant role in post-transcriptional processes, including alternative splicing and mRNA stability, acting as scaffolds or decoys for RNA-binding proteins and other RNAs [57]. These diverse mechanisms highlight lncRNAs' multifaceted role in cellular regulation, are versatile regulators of gene expression, extending far beyond mere transcriptional control. The luciferase assay results showed that the transcriptional activity of SRF enhanced by CARMN is dependent on CArG box in promoter regions of target VSMC contractile genes. It is also worth noting that CARMN alone did not promote the transcription of VSMC contractile marker genes, but the presence of SRF is necessary for CARMN to exert its function. In future studies, we aim to further unravel the specific pathways and molecular interactions through which CARMN orchestrates the expression of VSMC contractile genes, potentially uncovering novel therapeutic targets for vascular diseases.

To determine the critical role CARMN plays in AAA formation, we further analyzed the upstream mechanism that modulates the expression of CARMN. Interestingly, we predicted that NRF2, a master transcription factor in the antioxidant defense system, may interact with CARMN. NRF2 has been extensively studied in the context of inflammatory responses and oxidative stress through its regulation of the expression of genes encoding phase II detoxification enzymes and antioxidants [58, 59]. NRF2 has also been reported to regulate PDGF-stimulated VSMC migration. In this work, we found that NRF2 promoted the expression of CARMN, accompanied by an increase in VSMC contractile markers. Further luciferase assays also confirmed the binding of NRF2 to the promoter region of CARMN. Interference with NRF2 expression affects the regulatory effect of CARMN on the VSMC phenotype, which also provides evidence for the regulatory effect of NRF2 on CARMN. These results suggested that NRF2 can promote the transcription of CARMN through binding to its promoter region. A previous study reported that although the expression of NRF2 increases in human and mouse AAA tissues, overexpression of NRF2 can prevent Ang II-induced AAA development in mice [31]. Considering the protective role of NRF2, we propose that upregulated NRF2 serves as a self-protective mechanism for the body to maintain the expression of CARMN under pathological conditions.

Notably, there are some limitations in this study. First, although CARMN is enriched in VSMCs, we cannot completely ignore the small amount of expression in other types of cells. The use of AAV9 might intervene in the expression of CARMN in other cells, which would have an impact on VSMC function and AAA progression. Similarly, the presence of CARMN in SMCs of other organs or tissues, such as coronary artery SMCs and gastrointestinal SMCs, may also affect the experimental results. CARMN conditional knockout mice will provide evidence for its role in AAA formation in future studies. In exploring the functional role of CARMN in VSMC phenotypes transition, we didn’t perform a single-cell sequencing technology to further investigate the changes in the quantity and functionality of VSMCs induced by CARMN, as well as the potential types of VSMC transition. In the future, we plan to integrate single-cell sequencing technology to further explain the function of CARMN on VSMCs. In addition, CARMN has other downstream targets, while our study mainly focused on SRF. A complex mechanistic network by which CARMN regulates VSMC phenotypic transformation needs further clarification.

In summary, we discovered that CARMN protected against AAA formation and inhibited VSMC phenotypic transformation through interaction with SRF and enhanced its promotional effect on VSMC marker gene transcription. The expression of *Carmn* was enhanced by NRF2 binding to its promoter region. CARMN could serve as a therapeutic target for AAA treatment.

### Supplementary Information

Below is the link to the electronic supplementary material.

Supplementary file1 (XLSX 14 KB)

Supplementary file2 (DOCX 2596 KB)

Supplementary file3 (DOCX 14 KB)

Supplementary file4 (DOCX 14 KB)

Supplementary file5 (DOCX 14 KB)

Supplementary file6 (DOCX 14 KB)

Supplementary file7 (DOCX 17 KB)

## Data Availability

The data for this study are available by contacting the corresponding authors upon reasonable request.
