# Supplementary material for: LncRNA CARMN inhibits abdominal aortic aneurysm formation and vascular smooth muscle cell phenotypic transformation by interacting with SRF
Source: Cell Mol Life Sci. 2024 Apr 10;81(1):175. doi: 10.1007/s00018-024-05193-4 (PMC11006735; doi:10.1007/s00018-024-05193-4)

**Supplemental Figure I. Single-cell RNA sequencing analysis revealed that CARMN is enriched in VSMCs and participates in regulating VSMC contractile activity.** (A) A t-SNE plot shows all cells in the aortic samples from the sham-operated mice (Sham) and AAA samples from mice treated with PPE for 7 days (AAA_7d) and 14 days (AAA_14d). (B) A t-SNE plot shows all cells colored according to the 10 major cell types. (C) Heatmap shows the expression of marker genes of 10 cell types. (D) Bar plots show the cluster distributions of the 10 cell types in each group. (E) Dot plots show the relative expression of CARMN in each group (upper left); cells were projected onto a t-SNE plot. (F) Dot plots show the expression of CARMN in different cell types. (G) A t-SNE plot shows all VSMCs colored according to the 5 major cell types. (H) Heatmap shows the expression of marker genes of 5 VSMC types. (I) Dot plots show the expression of contraction markers (ACTA2, CNN1, MYH11 and TAGLN) in 5 VSMC types. (J) Violin plots show the score of VSMC contraction in 5 VSMC types according to the AddModuleScore (left). The cells were projected onto a t-SNE plot (right). (K) Dot plots show the expression of CARMN in VSMCs in different groups (left); cells were projected onto a t-SNE plot (right).

**
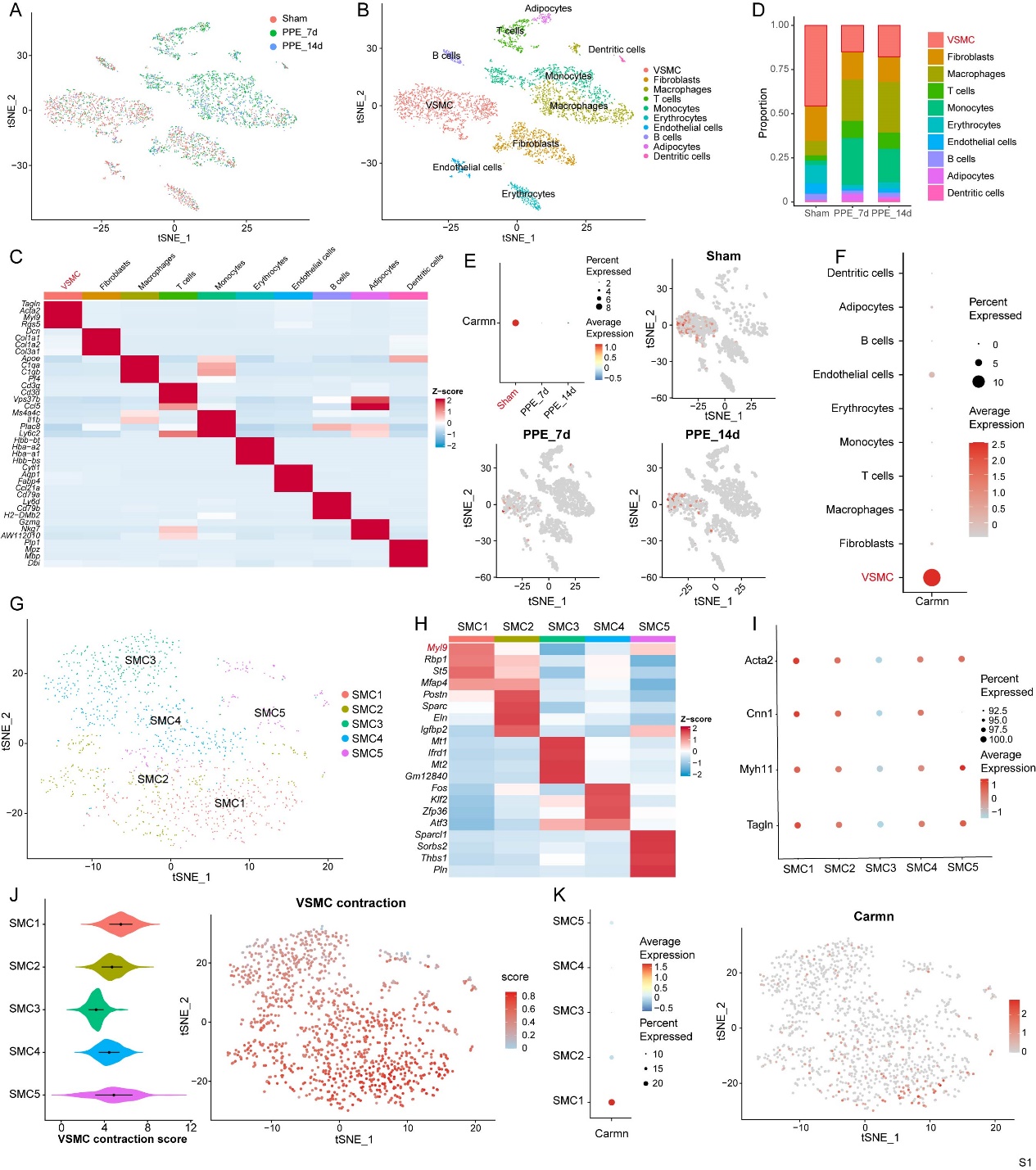
**

**Supplemental Figure II.** (A) The expression of mRNAs in each group after the raw data were standardized. (B) PCA of mRNAs between PPE-induced AAA samples and the control samples. (C) The expression of ncRNAs in each group after the raw data were standardized. (D) PCA of ncRNAs between PPE-induced AAA samples and control samples.

**
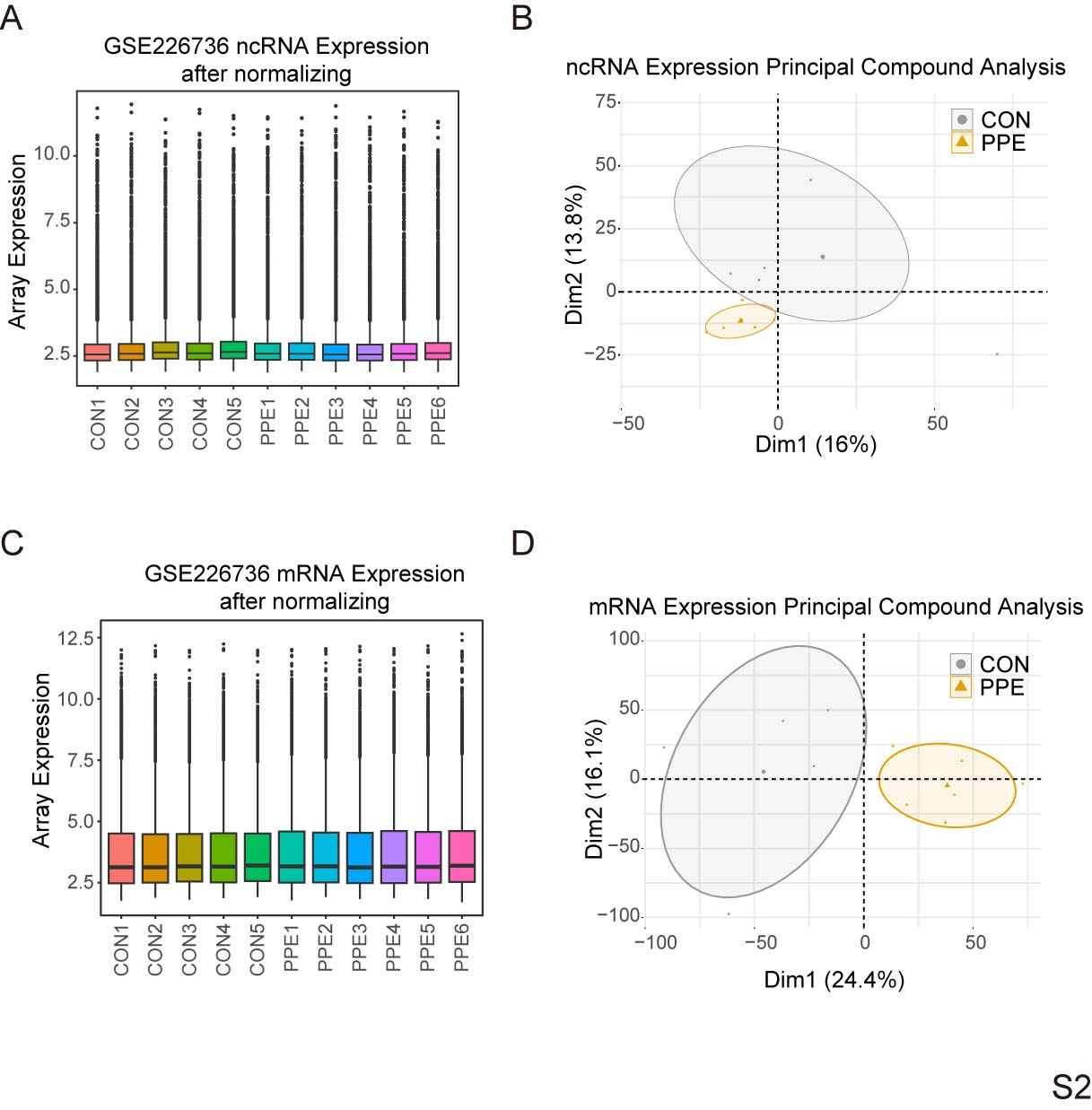
**

**Supplemental Figure III.** (A) Volcano plot shows the differential expression of mRNAs between PPE-induced AAA samples and control samples. (B) Heatmap shows the differential expression of mRNAs in each group. (C) to (E) GO analysis of differentially expressed mRNAs. (F) KEGG analysis of differentially expressed mRNAs.

**
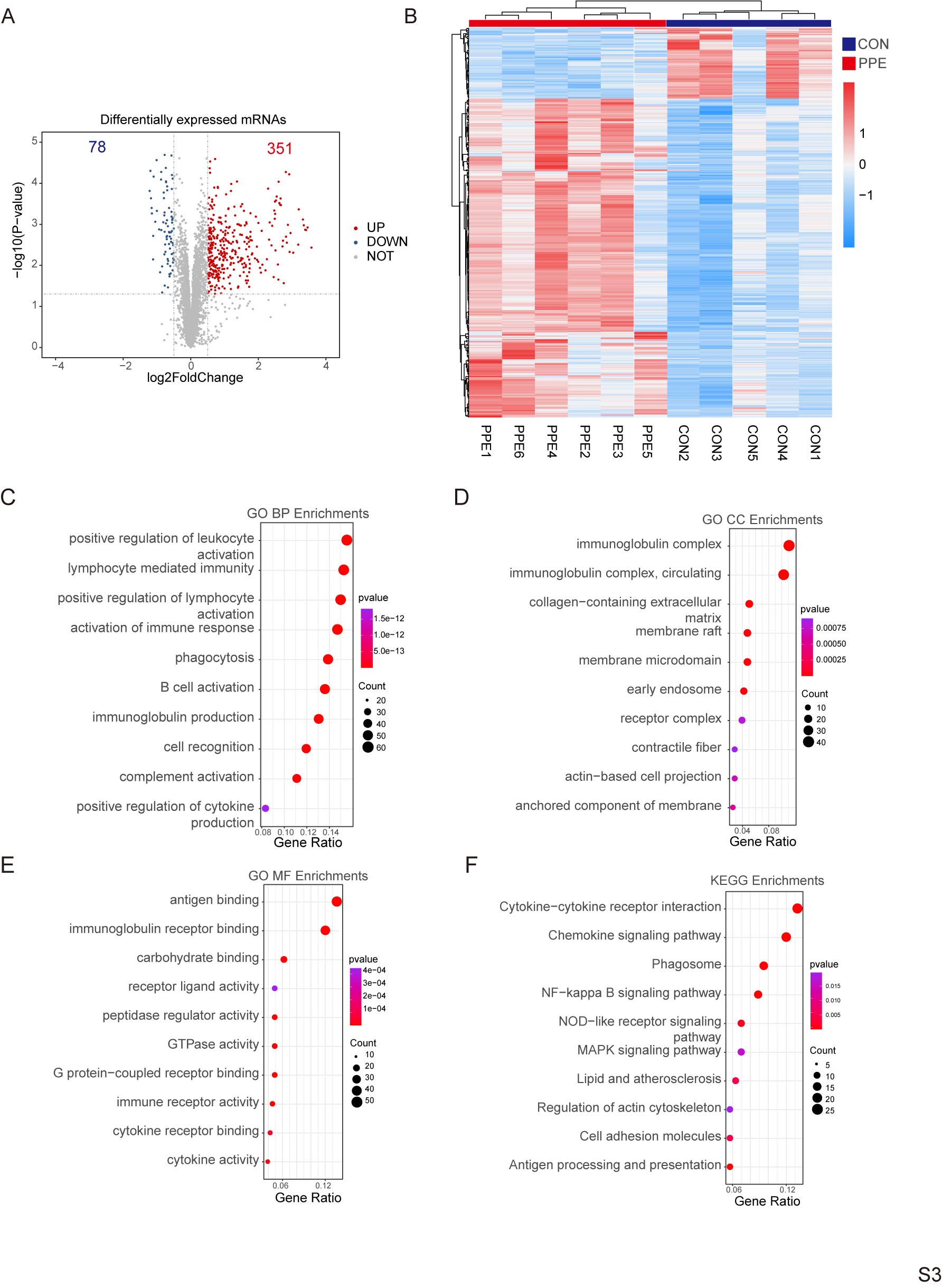
**

**Supplemental Figure IV.** (A) The expression of mRNAs in each group after the raw data were standardized. (B) PCA of mRNAs between the aneurysm and control groups. (C) The expression of ncRNAs in each group after the raw data were standardized. (D) PCA of ncRNAs between the aneurysm and control groups. (E) Volcano plots show the differential expression of ncRNAs between the aneurysm and control groups. (F) Heatmap shows the differential expression of ncRNAs in each group. (G) The differential expression of Carmn between the aneurysm and control groups (****p=9.5e-08).


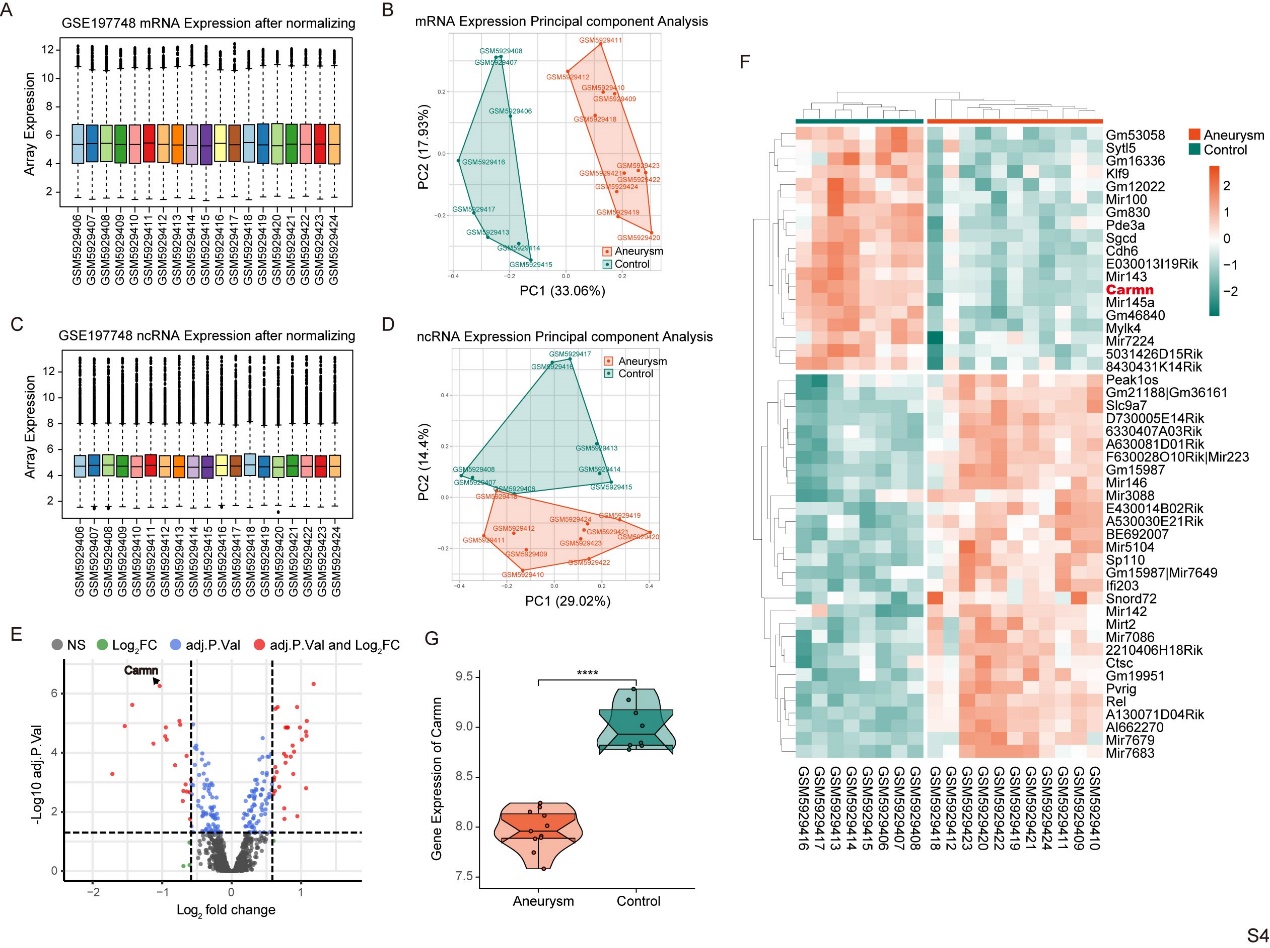


**Supplemental Figure V.** (A) to (C) GO analysis of differentially expressed mRNAs. (D) KEGG analysis of differentially expressed mRNAs.

**
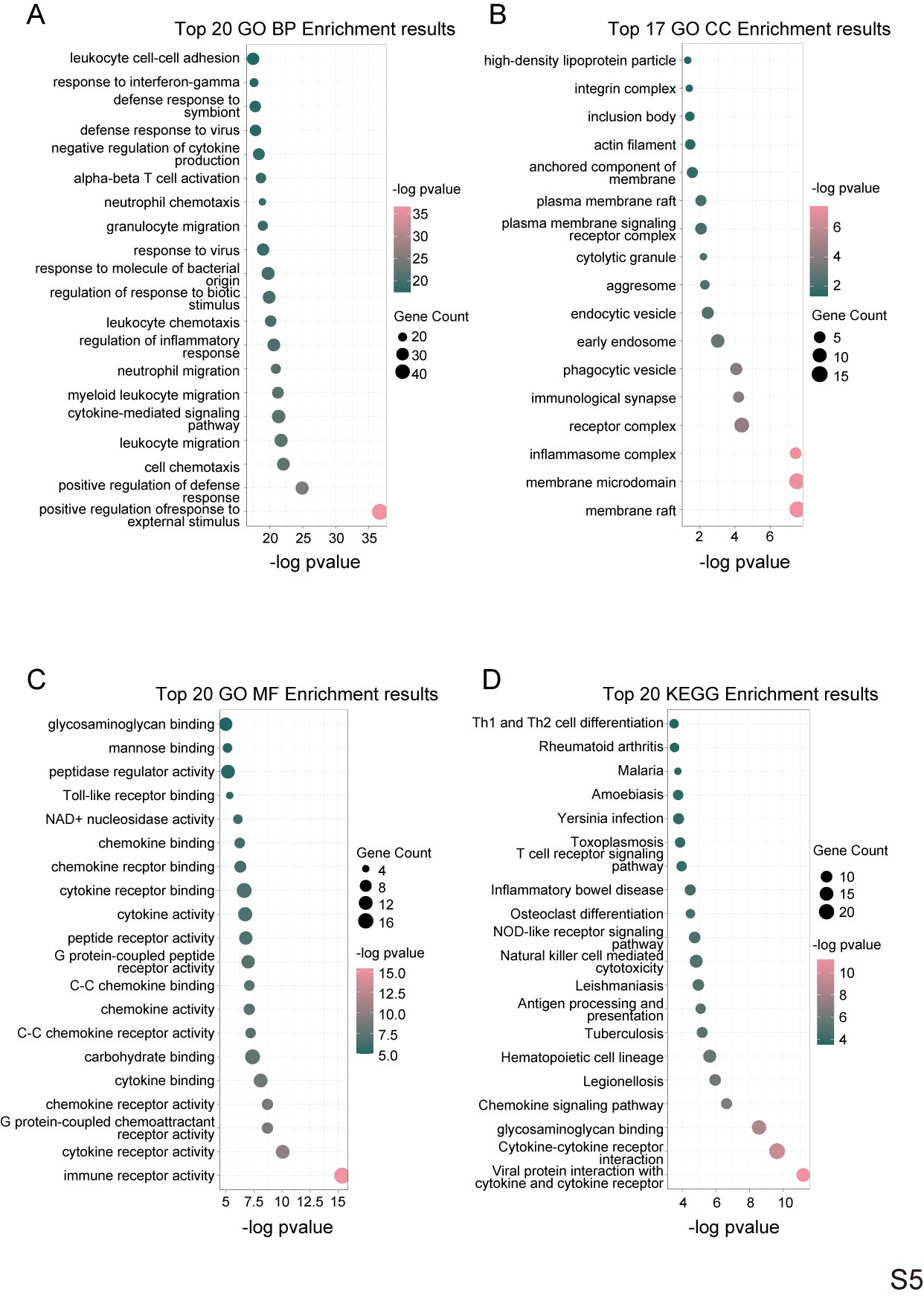
**

**Supplemental Figure VI.** (A) Differential expression of mRNAs coexpressed with Carmn in each group. (B) to (D) GO analysis of mRNAs coexpressed with Carmn. (E) KEGG analysis of mRNAs coexpressed with Carmn. (F) Metascape analysis of mRNAs coexpressed with Carmn. (G) GeneMANIA analysis showing the PPI network of mRNAs coexpressed with Carmn.


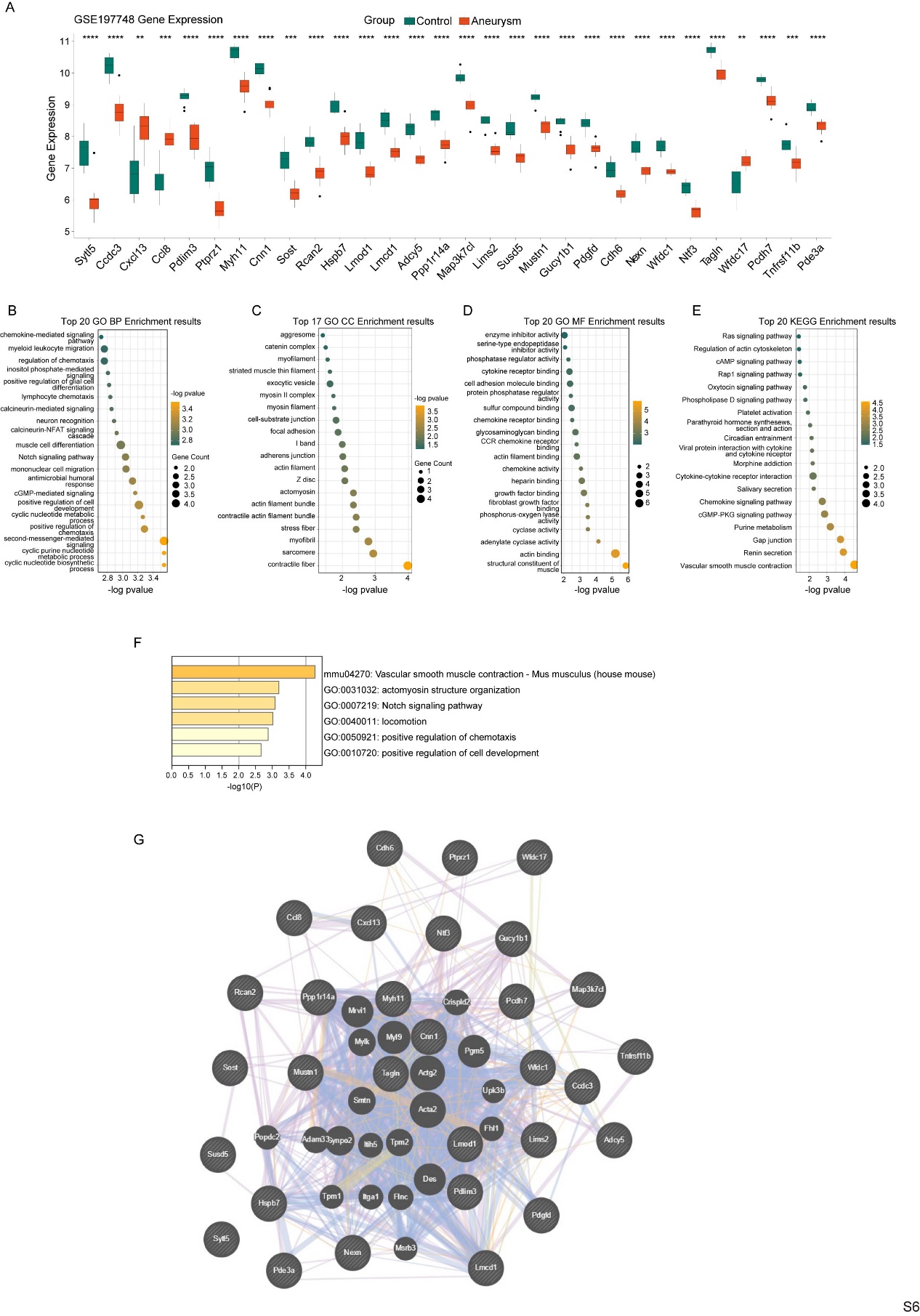


**Supplemental Figure VII.** Relative CARMN levels in the aorta, heart, lung, liver, brain and skeletal muscle of mouse (**p<0.01, n=5 in each group).

**
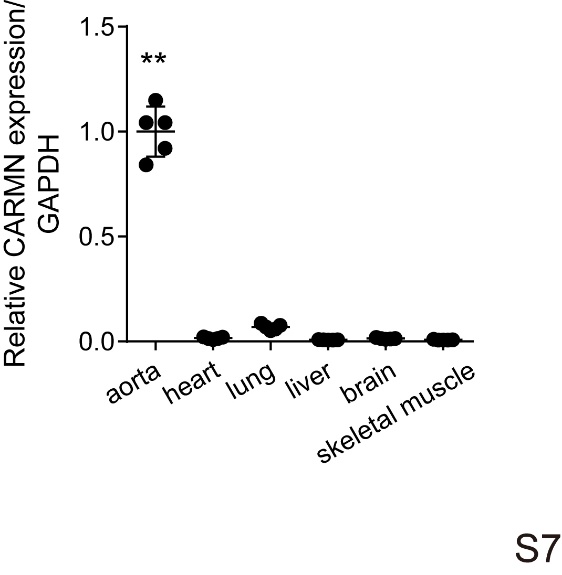
**

**Supplemental Figure VIII.** (A) Schematic graph of AAV transfection and PPE-induced AAA model establishment. (B) Immunofluorescence staining of GFP (green), SM22α (red) and DAPI (blue) in mouse abdominal aortas 30 days after transfection with Scr-RNA or Sh-CARMN. (C) Relative CARMN levels in mouse abdominal aortas 30 days after transfection with Scr-RNA or Sh-CARMN (***p*<0.01, n=5 in each group). (D) Relative mRNA levels of α-SMA, CNN1 and SM22α detected by qPCR in the abdominal aortas from Ang II–infused mice in each group (***p*<0.01, n=5 in each group).

**
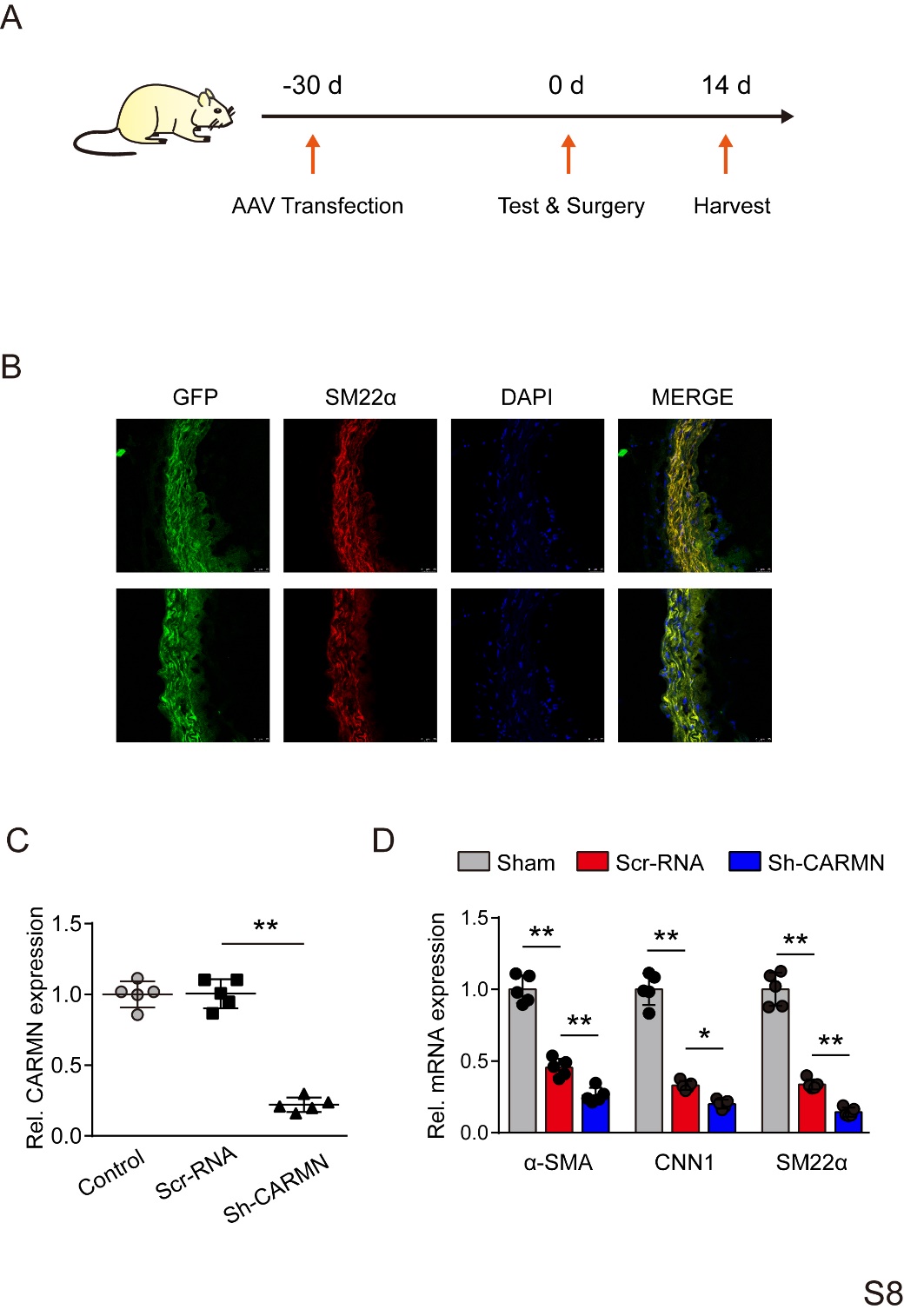
**

**Supplemental Figure IX.** (A) Immunofluorescence staining of GFP (green), SM22α (red) and DAPI (blue) in mouse abdominal aortas 30 days after transfection with AAV-GFP or AAV-CARMN. (B) Relative CARMN levels in mouse abdominal aortas 30 days after transfection with AAV-GFP or AAV-CARMN (***p*<0.01, n=5 in each group). (C) Relative mRNA levels of α-SMA, CNN1 and SM22α detected by qPCR in the abdominal aortas from Ang II–infused mice in each group (***p*<0.01, n=5 in each group).


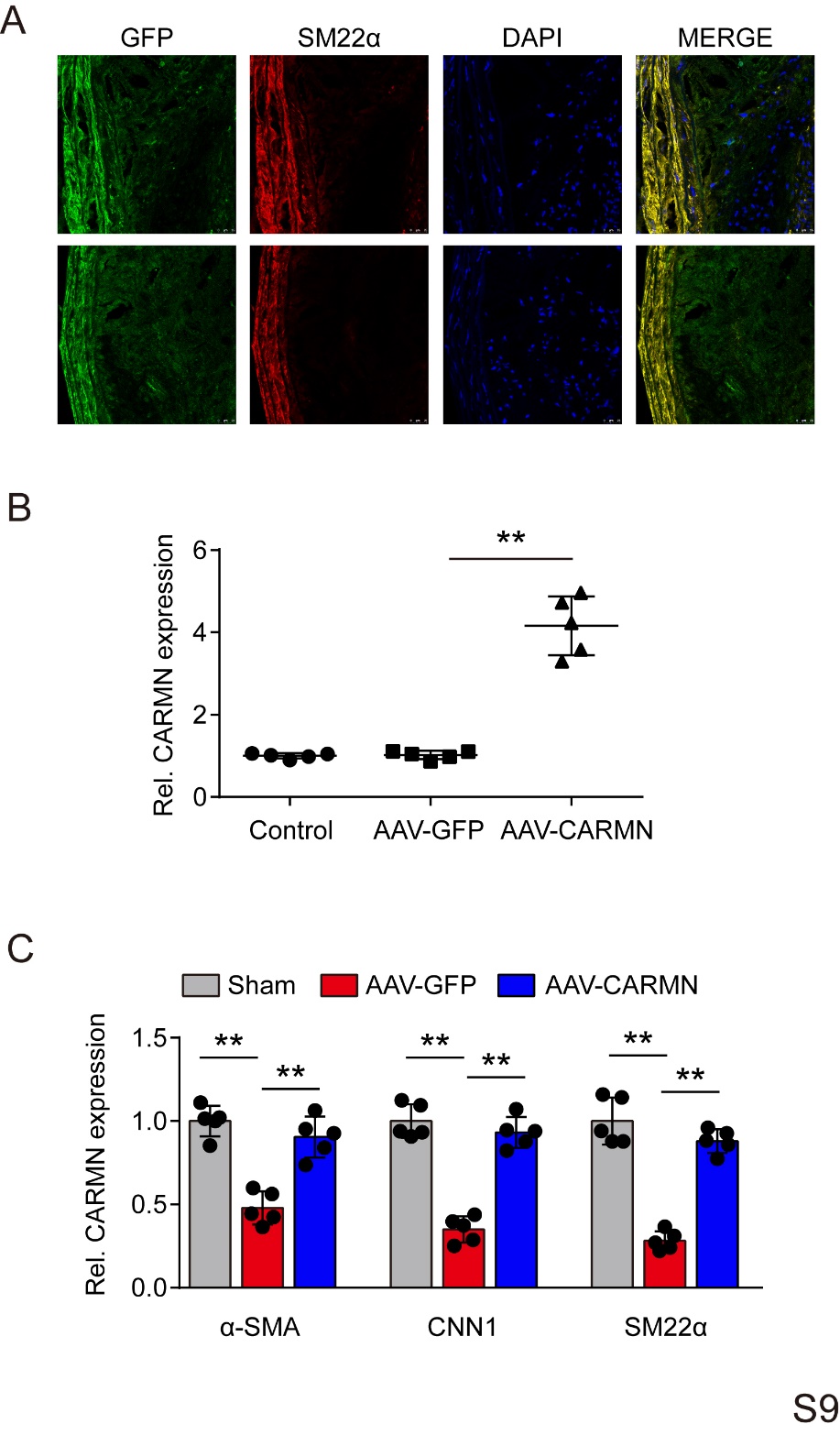

Supplement: Supplementary file 2 — Supplementary file2 (DOCX 2596 KB) [file 18_2024_5193_MOESM2_ESM.docx]
