# Supplementary material for: LncRNA CARMN inhibits abdominal aortic aneurysm formation and vascular smooth muscle cell phenotypic transformation by interacting with SRF
Source: Cell Mol Life Sci. 2024 Apr 10;81(1):175. doi: 10.1007/s00018-024-05193-4 (PMC11006735; doi:10.1007/s00018-024-05193-4)
Supplement: Supplementary file 3 — Supplementary file3 (DOCX 14 KB) [file 18_2024_5193_MOESM3_ESM.docx]

**Supplemental Table 1. Quantitative real-time PCR primer sequences.**

| Primer | Sequence (5'-3') |
| --- | --- |
| GAPDH (mouse) | Forward: TGTCCGTCGTGGATCTGAC  Reverse: CCTGCTTCACCACCTTCTTG |
| CARMN (mouse) | Forward: GACAACCAGTACCAAGAAGAGTGCC  Reverse: CCTAGAACCTGCCGATGACTTTGCA |
| α-SMA (mouse) | Forward: TCCTGACGCTGAAGTATCCGAT  Reverse: GGCCACACGAAGCTCGTTATAG |
| CNN1 (mouse) | Forward: GCGTCACCTCTATGATCCCAA  Reverse: CCCAGACCTGGCTCAAAGAT |
| SM22α (mouse) | Forward: GATATGGCAGCAGTGCAGAG  Reverse: AGTTGGCTGTCTGTGAAGTC |
| GAPDH (human) | Forward: ACGGATTTGGTCGTATTGGG  Reverse: TGATTTTGGAGGGATCTTGC |
| CARMN (human) | Forward: AATTAGTTGAGAAGCAGTGACACC  Reverse: CAGAGTTCTTGCTTCTCTGACATC |
| α-SMA (human) | Forward: GACCTTTGGCTTGGCTTGTC  Reverse: GTGCGGACAGGAATTGAAGC |
| CNN1 (human) | Forward: TTAAGAACAAGCTGGCCCAGAAGT  Reverse: AAGTTGCCGATGTTCTCCAGC |
| SM22α (human) | Forward: TCAAGCAGATGGAGCAGGTG  Reverse: CCACGGTAGTGCCCATCATT |
