# Supplementary material for: LncRNA CARMN inhibits abdominal aortic aneurysm formation and vascular smooth muscle cell phenotypic transformation by interacting with SRF
Source: Cell Mol Life Sci. 2024 Apr 10;81(1):175. doi: 10.1007/s00018-024-05193-4 (PMC11006735; doi:10.1007/s00018-024-05193-4)
Supplement: Supplementary file 4 — Supplementary file4 (DOCX 14 KB) [file 18_2024_5193_MOESM4_ESM.docx]

**Supplemental Table 2. Antibodies for western blotting.**

| Antibody | Vendor or Source | Catalog # | Dilute Proportion |
| --- | --- | --- | --- |
| anti-α-SMA | Abcam | ab32575 | 1/1000 |
| anti-CNN1 | Abcam | ab46794 | 1/5000 |
| anti-SM22α | Abcam | ab14106 | 1/1000 |
| anti-EIF5 | Abcam | ab228874 | 1/1000 |
| anti-SRF | Abcam | ab252868 | 1/1000 |
| anti-GAPDH | Abcam | ab181602 | 1/10000 |
| anti-NRF2 | Proteintech | 80593-1-RR | 1:1000 |
