# Supplementary material for: LncRNA CARMN inhibits abdominal aortic aneurysm formation and vascular smooth muscle cell phenotypic transformation by interacting with SRF
Source: Cell Mol Life Sci. 2024 Apr 10;81(1):175. doi: 10.1007/s00018-024-05193-4 (PMC11006735; doi:10.1007/s00018-024-05193-4)
Supplement: Supplementary file 5 — Supplementary file5 (DOCX 14 KB) [file 18_2024_5193_MOESM5_ESM.docx]

**Supplemental Table 3. Antibodies for immunofluorescence.**

| Antibody | Vendor or Source | Catalog # | Dilute Proportion |
| --- | --- | --- | --- |
| anti-α-SMA | Abcam | ab32575 | 1/100 |
| anti-SM22α | Abcam | ab14106 | 1/100 |
| anti-GFP | Abcam | ab13970 | 1/2000 |
