# Supplementary material for: LncRNA CARMN inhibits abdominal aortic aneurysm formation and vascular smooth muscle cell phenotypic transformation by interacting with SRF
Source: Cell Mol Life Sci. 2024 Apr 10;81(1):175. doi: 10.1007/s00018-024-05193-4 (PMC11006735; doi:10.1007/s00018-024-05193-4)
Supplement: Supplementary file 6 — Supplementary file6 (DOCX 14 KB) [file 18_2024_5193_MOESM6_ESM.docx]

**Supplemental Table 4. CARMN probe sequences.**

| CARMN probe | Sequence |
| --- | --- |
| Human | atgtctctggctgtgggggacctctgtcttctgttgggaggcttggagaggcgtgggtga  gagttctgtctccgggctgcctgctgctggccctcctctggtcccaggaggctgcttctc  cagagttcttgcttctctgacatcagcatggcggggtggctcccaccagctagcgccctt  gcctgtgcacttgttacagccgttgctctccttgggctccagggccgtagggtttccacc  acatgtctttcctggggtctggtccaggtgtggctccttggggcctggagacgggagcca |
| Mouse | gtgtgtgcacgagtgtgtgtgtgtgtgtgactacagacaaaagatgacacgtggtgacca  tcttctgtggccacggcttgtgtgggaacttcggggtgaacagaacacccctgaccccca  aaccacctactcaccctgctcttgctaagacacccccctcccccagggtccccagataac  ctttgcttcgtggtgactgcggtggccttggtgatggatgatacataccttcagggtttt  ctgaagagggtaggcagcctttcctcagggctggcactct |
