# Supplementary material for: LncRNA CARMN inhibits abdominal aortic aneurysm formation and vascular smooth muscle cell phenotypic transformation by interacting with SRF
Source: Cell Mol Life Sci. 2024 Apr 10;81(1):175. doi: 10.1007/s00018-024-05193-4 (PMC11006735; doi:10.1007/s00018-024-05193-4)
Supplement: Supplementary file 7 — Supplementary file7 (DOCX 17 KB) [file 18_2024_5193_MOESM7_ESM.docx]

**Supplemental Table 5. Patient clinical information.**

| Patient | Gender | Age | Smoking status | Diameter (mm) | HL | HTN | CAD |
| --- | --- | --- | --- | --- | --- | --- | --- |
| ID1 | male | 65 | yes | 60 | yes | yes | yes |
| ID2 | male | 71 | yes | 63 | yes | yes | no |
| ID3 | male | 64 | no | 58 | yes | yes | no |
| ID4 | male | 59 | yes | 63 | yes | yes | yes |
| ID5 | male | 62 | yes | 59 | yes | yes | yes |

HL = Hyperlipidemia

HTN = Hypertension

CAD = Coronary artery disease
